# Evaluation of Connectivity Reliability of VANETs Considering Node Mobility and Multiple Failure Modes

**DOI:** 10.3390/s25196073

**Published:** 2025-10-02

**Authors:** Junhai Cao, Yunlong Bian, Chengming He, Fusheng Liu, Dan Xu, Yiming Guo

**Affiliations:** 1 Department of Equipment Support and Remanufacturing, Army Academy of Armored Forces, Beijing 100072, China; jhcao@sina.com (J.C.); chengminghe@sina.com (C.H.);; 2 National Key Laboratory of Intelligent Parallel Technology, Beijing 100072, China; 3 Unit 92942, PLA, Beijing 100161, China; yimingguo@sina.com

**Keywords:** VANETs, connectivity reliability, mobility, failure, simulation

## Abstract

As a subclass of Mobile Ad hoc Networks (MANETs), Vehicle Ad hoc Networks (VANETs) possess multi-hop relay communication and dynamic topology reconstruction capabilities and are widely applied in various social activities. When they are used as clusters to perform various disaster search and rescue operations or communication relay, reliable, secure, and timely communication connectivity becomes particularly important. This paper focuses on the research of connectivity reliability in VANETs, emphasizing the impact of node movement characteristics and various failure modes on the connectivity reliability of VANETs: As a cluster, the nodes in VANETs have interactive relationships and no longer follow a random movement model, exhibiting regular movements of the network as a whole; the failure modes of nodes in VANETs include vehicular hardware/software failure, energy consumption failure, intentional attack, and isolation failure. Additionally, to optimize node communication energy consumption, the paper proposes a routing path identification algorithm. Finally, the paper presents a simulation algorithm for solving the connectivity reliability of VANETs. Through MATLAB simulation experiments, the effectiveness and correctness of the proposed algorithm are verified, and it is found that the attraction distance between nodes has a certain impact on the isolation failure mode and connectivity reliability.

## 1. Introduction

The recent advancements in wireless communication technology and the Internet of Things, coupled with the urgent need for interconnectivity and enhanced transportation efficiency in the real world, have propelled the rapid development of vehicle ad hoc networks (VANETs) [1,2,3,4]. As a type of Infrastructure-Less Network (ILN), VANETs can leverage their self-organizing capabilities to flexibly and swiftly configure the network to support communication for specific tasks, all while minimizing the need for infrastructure. The vehicle-to-vehicle (V2V) communication function of VANETs not only plays a significant role in urban traffic management, enabling functions such as emergency broadcasting and collision avoidance for road vehicles, but also assists managers in scenarios such as fire rescue, earthquake rescue, and flood rescue, as well as the exploration of unknown forest environment [5]. In the latter cases, VANETs are often utilized in a cluster form [6]. During the deployment phase, the overall mission objectives and scope of the VANETs are clearly defined. During the mission execution phase, reliable connectivity within the network is achieved through V2V and vehicle-to-infrastructure (V2I) communication, which continuously constraining individual node behaviors and adjusting cluster behaviors.

As a special type of engineering system, a VANET has nodes that can be further abstracted as a collection of hardware devices (such as on-board units) and software. Consequently, nodes possess general quality characteristic indicators such as reliability. When hardware or software malfunctions, it may affect the communication connectivity between nodes, even leading to some nodes going offline. The energy of nodes in VANETs also has a certain impact on communication functions. When there is no energy replenishment during the execution of tasks in VANETs, the greater the demand for node information transmission and reception, the direct increase in information processing volume, and thus, accelerated energy consumption. When the node energy is insufficient to support information reception or transmission functions, the node will lose its communication function [7]. This situation is more prominent in clusters such as autonomous vehicle fleets and wireless sensor networks (WSNs). With the continuous exploration of VANETs by engineering and management personnel, VANETs are increasingly active in certain hazardous application scenarios, such as fire rescue, and emergency communication networking in earthquake disaster areas. Such scenarios often have an obvious hazard source (such as a fire point, or epicenter) and form a hazardous area with the hazard source as the radius. When VANETs enter the hazardous area and move closer to the hazard source, the probability of node malfunctions due to high temperatures, aftershocks, and human attacks increases significantly. Moreover, in urban application scenarios of VANETs, there are also deliberate attack events such as Denial of Service (DoS) and identity spoofing [8,9,10,11]. For example, an attacker sends a large amount of beacon information at an intersection, preventing other vehicles from receiving real traffic information [3,12]. The paper collectively refers to these as intentional attack events [13]. As a special type of network system, VANETs have nodes that also exhibit a special failure mode during mutual communication, isolation failure. When a node in VANETs has both hardware/software working normally, sufficient remaining energy, and is not subject to intentional attacks, if all neighboring nodes within its communication coverage lose communication functionality due to various failure events, the node is in an “offline” state as it cannot establish communication connectivity with any node in VANETs, and thus will no longer exert any impact on the VANET. In addition, when VANETs operate in a cluster form, the connections between nodes are closer, and there is often a control relationship [14,15]. Each node continuously sends and receives information related to the overall movement direction of the network, road conditions, node coordinates, and network task details under the influence of one or a few control nodes. While ensuring the overall movement consistency of VANETs as a cluster, the movement of each network node exhibits a certain degree of randomness. In general traffic management scenarios, vehicle movement often exhibits significant randomness [16], and the connections between different nodes are not tight. This paper will investigate the impact of the overall motion characteristics of the network on the connectivity reliability of VANETs [6].

Abstracting vehicles with communication functions in VANETs as vertices or nodes, and the connections between vehicles as edges or arcs, we can obtain the general graph theory model of VANETs $G=\left(V,E\right)$, where $V=\left\{N_{1},N_{2},\ldots ,N_{m}\right\}$ is the set of vertices, representing m vehicles; $E=\left\{e_{ij}|N_{i},N_{j}\in V;i\neq j\right\}$ is the set of edges, representing communication links between different vehicles, such as $e_{ij}$ indicating that there is a link between node $N_{i}$ and $N_{j}$. The model is determined at the deployment stage and task initiation of VANETs. Analogizing the relationship between components and systems in classical reliability theory, nodes, as the basic components of the network system, are the foundation of VANETs. Combining domestic and international research, in current studies on the connectivity reliability of VANETs, it is often assumed that network nodes are idealized perfect nodes [17,18], meaning that nodes do not fail or malfunction and will always be in working condition during the task time. However, in reality, VANETs nodes composed of communication devices or equipment have basic reliability and other quality characteristics, and their hardware or software may fail, which in turn affects communication quality and even communication connectivity. Some studies consider non-perfect nodes [19], meaning that nodes may malfunction, but they assume that failures between nodes are independent, ignoring the correlation between node failures. This paper defines VANETs nodes as mobile vehicles with communication functions and makes the following assumptions:

(1) VANETs nodes are non-perfect nodes [20], which exhibit fault modes and fault states.

(2) VANETs nodes possess both communication and mobility functions. They can be abstracted as n×n points on a two-dimensional coordinate plane, and their positions are determined by $\left(x,y\right)$ coordinates. The core function of VANETs nodes studied in this paper is the communication function, without considering the scenario where the mobility function fails, i.e., there is no mobility failure mode or mobility function failure state, to focus on the analysis and evaluation of VANETs connectivity reliability [21].

(3) VANETs nodes are categorized into key control nodes ($N^{C}$) and general mobile nodes ($N^{M}$) based on their command relationships [5,6,22].

(4) Any nodes in VANETs can be interconnected without relying on fixed relay nodes or infrastructure, provided that the communication requirements are met. There is no signal interference between VANETs nodes [2,23,24].

(5) The wireless links in VANETs are symmetric and fault-free, adhering to a binary model: if the distance between nodes is less than the communication distance threshold, a communication link can be established [21].

(6) The battery within VANETs nodes is non-rechargeable and has limited energy [21,25].

(7) The paper selects the shortest path routing protocol [22,26,27] to support the information transmission and reception activities of VANETs nodes: that is, information is transmitted only along the shortest path between each pair of nodes, and the path consists of connected links between nodes, with the path length equal to the number of links.

Based on the assumptions, the paper comprehensively considers random node failures, correlations between nodes, node energy depletion, and mobility characteristics to derive a node failure model and establish a node reliability model. The failure events of VANETs nodes mainly include four categories: hardware/software failure, energy consumption failure [25], intentional attack, and isolation failure. Denoted S as the node state set, each node has five states $S=\left\{1,2,3,4,5\right\}$: S=1 represents that the node’s communication function is normal and it can complete information transmission and reception tasks with neighboring nodes; S=2 represents that the node has experienced a hardware/software failure event, lost its communication function, and is in a failed state; S=3 represents that the node has experienced an energy depletion event, lost its communication function, and is in a failed state; S=4 represents that the node has suffered an intentional attack, lost its communication function, and is in a failed state; S=5 represents that the node cannot connect with other nodes in the network, lost its communication function, and is in an isolation failure state. It is stipulated that only nodes that meet the specified criteria are considered normal nodes and can achieve communication functions. Nodes in other states will maintain their corresponding failure states after experiencing a failure event, and there is no transition between different failure states, meaning that a node only experiences one type of failure.

Compared with existing research, the research in this paper primarily focuses on the cluster application scenarios of VANETs, comprehensively considering the overall movement trend of the network, task objectives, and the impact of multiple failure modes of network nodes on the connectivity reliability of VANETs. Firstly, mathematical analysis and modeling are conducted for four failure modes existing in VANETs nodes. The energy consumption failure model can be further extended to the reliability analysis of unmanned vehicle clusters and wireless sensor networks. The intentional attack failure mainly considers various external attacks that may be encountered during the execution of dangerous tasks in VANETs. Secondly, the paper proposes an improved Couzin-leader model to describe the interaction relationships and movement behaviors of nodes within VANETs in a cluster form, highlighting the overall characteristics of the network. Subsequently, a routing path identification algorithm is proposed for the periodic information transmission scenario of VANET nodes, with the goal of reducing the amount of repeated information transmission and energy consumption. Finally, a simulation algorithm for solving the connectivity reliability of VANETs is proposed and implemented in MATLAB. The research is validated through parameter indicators and sensitivity analysis. The main contributions of the paper include:

- Comprehensively considering four types of failure modes: hardware/software failure, energy consumption failure, intentional attack, and isolation failure, we model the nodes in VANETs, thereby expanding the versatility of the node failure model;
- Design a cluster application scenario for VANETs, considering factors such as the overall network objectives, the influence relationships between nodes, the movement trends of the network and nodes, and periodic information transmission;
- Design a simulation algorithm and metrics for solving the connectivity reliability of VANETs, and further investigate the impact of attraction distance between nodes on node failure and network connectivity reliability in cluster application scenarios of VANETs through sensitivity analysis.

## 2. Related Work

### 2.1. Research on the Reliability of Vehicular Ad Hoc Networks

Many scholars have studied the connectivity reliability of VANETs in urban environments, and expanded from node failure analysis to link communication quality, channel analysis, etc. Dharmaraja et al. [20] focused on VANETs as their research subject. Given the crucial importance of reliability and survivability to road safety, and the insufficient attention previously paid to the impact of hardware and channel availability, they adopted hierarchical modeling techniques. Utilizing reliability block diagrams, Markov chains, and Markov reward models, they conducted research on V2V and V2R (Vehicle-to-Roadside) communication. They analyzed the reliability of on-board units (OBUs) and roadside units (RSUs), network reliability, channel availability, message transmission, connectivity, and other related aspects. The validity of their model was verified through MATLAB Simulink simulations. They analyzed the trends of network reliability, connectivity, and other indicators as they varied with factors such as time and the number of nodes. Shelly et al. [28] proposed a next-hop node selection scheme based on the residual lifetime of links to address the issue in VANETs where traditional greedy forwarding strategies fail to consider link reliability, often leading to transmission failures. This scheme utilizes Kalman filtering to predict the residual lifetime of links between vehicles. By constructing a system model to describe vehicle movement and communication, and combining the Gaussian-Markov mobility model to analyze vehicle relative speed and position, it selects the node corresponding to the link with the longest residual lifetime as the forwarding node. Regragui et al. [29] focused on the impact of intelligent mobility design on the connectivity dynamics of VANETs in urban environments. They proposed three mobility models based on Cellular Automata (CA) (real-time path update, fixed path, traffic light control), combined with the Bellman-Ford algorithm to implement intelligent path planning, and simulated real traffic scenarios such as roundabout traffic rules and vehicle acceleration and deceleration behaviors. Through NS-2 simulation, they analyzed the impact of factors such as vehicle density, transmission range, and RSU deployment on traffic flow, average speed, packet delivery rate, end-to-end delay, and other indicators.

Babu et al. [30] propose a distributed adaptive multicast routing protocol to address the challenges of multicast tree construction and maintenance caused by high mobility and dynamic topology in vehicle ad hoc networks. This protocol dynamically selects high-stability parent nodes and guardian nodes, utilizes link expiration time to quantify link stability, achieves efficient construction of multicast trees and rapid switching in case of failure, and reduces network fragmentation and reconnection delay. Kadhim et al. [31] propose a delay-efficient multicasting scheme, DMPFS, based on parked vehicles, fog computing, and software-defined networking (SDN), to address the challenges of high latency and unstable links faced by secure information multicasting in vehicular ad hoc networks. By integrating parked vehicles as fixed fog nodes into a four-layer network architecture (vehicle layer, FC layer, OpenFlow switch layer, and SDN controller layer), DMPFS leverages the centralized control capabilities of SDN and the edge processing capabilities of FC, combined with bandwidth-constrained optimization models, priority scheduling strategies, and partitioning techniques, to construct low-latency multicast routes and dynamically manage links and sessions.

Sohail et al. [32] comprehensively reviewed the routing protocols in VANETs, focusing on their key roles in intelligent urban transportation systems and challenges such as high mobility and sparse connectivity. Through systematic classification, they analyzed in detail the working principles, architectures, and application scenarios of topology-based, location-based, anycast, multicast, and broadcast-based protocols. Combining standardized achievements such as IEEE 802.11p, they compared their performance metrics (such as packet delivery ratio, delay, and routing overhead) and pointed out deficiencies. The study also summarized the applicable scenarios of simulation tools such as NS-2, MATLAB, and QualNet. Finally, it looked forward to future directions, including integrating 5G and machine learning to optimize routing decisions, improving the efficiency, reliability, and security of protocols in dynamic scenarios, and providing theoretical support for the practical deployment of VANETs in intelligent transportation systems.

Dui et al. [33] took the unmanned vehicle distribution network in the Internet of Things (IoT) environment as the research object, constructed a network model, analyzed the cascading failure process, and proposed two shunting methods based on importance; established a resilience model, defined performance indicators and resilience calculation methods, and proposed three optimization strategies; designed a Floyd time-varying resilience optimization algorithm.

### 2.2. Research on the Reliability of Similar Self-Organizing Networks

Considering that VANETs are a subclass of MANETs, the connectivity reliability evaluation of similar networks is also valuable for the research of this paper. Fu et al. [34] proposed a comprehensive fault model based on CA for binary WSNs, addressing complex fault issues driven by multiple factors such as energy exhaustion, hardware/software malfunctions, and impaired connectivity. The model systematically evaluates the invulnerability of WSNs under three strategies: random attacks, maximum-degree attacks, and maximum-betweenness attacks, and explores the impact of sink node layouts (random, degree-oriented, and betweenness-oriented).

Xiang et al. [27] focused on the research of performance reliability in mobile ad hoc networks and defined transmission reliability to measure its transmission performance. By setting up network, node mobility, transmission, and interference models, they constructed a performance reliability evaluation method. However, in their study, all nodes moved within the network area according to a Random Direction Mobility Model (RDMM) with a constant speed, simplifying the complex mobility characteristics of nodes; and they did not describe the impact of node positions at specific time points on network reliability, lacking time-related and location-related reliability indicators.

Kumar et al. [35] proposed a Free Space Two Ray Ground Propagation (FS-TRG) hybrid model to assess network performance, such as connectivity, during task execution, addressing the connectivity issues in MANETs caused by node mobility. In their study, the Random Way Point Mobility Model (RWPM) was employed to mathematically describe the distribution of mobile nodes, and link reliability was determined based on the hybrid model. MATLAB simulations were conducted by setting various simulation parameters to analyze the impact of propagation parameters and network size on path reliability and average hop count. The research findings indicate that due to the characteristics of signal propagation, the reliability of homogeneous MANETs may decrease. Nevertheless, even with lower reliability values, the network can still maintain a certain level of reliability, providing a reference for users to deploy MANETs in suitable scenarios. However, the study assumed that node positions are uniformly and randomly distributed within the coverage area, and the network is homogeneous, with different nodes having the same reliability, ignoring factors such as the heterogeneity and non-uniform distribution of actual mobile networks.

Wang et al. [21] took wireless sensor networks as the research object. Addressing the issue that existing research on transmission reliability evaluation often overlooks the impact of multiple failure modes and random factors, they proposed a node reliability model that comprehensively considers random failures and energy consumption failures, a Signal-to-Noise Ratio-Capacity (SNR-Capacity) connection model that takes into account the randomness of channel capacity, as well as a transmission reliability evaluation model and algorithm based on the Sum of Disjoint Products (SDP) method and Monte Carlo simulation. Through case analysis, the effectiveness of the models and algorithms was verified. Furthermore, the study explored the impact of factors such as channel capacity randomness, energy consumption failures, transmission periods, transmission power, and background noise power on transmission reliability, providing a basis for accurately evaluating transmission reliability. However, the algorithms and methods proposed in the study were only applied to static networks with fixed topologies and did not consider possible mobility characteristics.

The relevant literature is further compared with this paper through Table 1.

## 3. Analysis and Modeling of Node Hardware/Software Failure

The implementation of communication functions in VANET nodes primarily relies on various communication devices (such as OBUs) and their software systems [36]. Among these, hardware devices exhibit basic reliability parameters such as MTTF and adhere to certain failure patterns [19,34]. Drawing from existing research literature [21], this paper notes that various hardware and software components within VANETs often experience sporadic failures. Therefore, the paper assumes that hardware/software failure events in VANETs nodes follow an exponential distribution and models them as follows:

(1) $$\mathrm{Pr}\left(S_{N_{i}}\left(t\right)=2\right)=1-e^{-\lambda_{i}t}$$

In the formula, $S_{N_{i}}\left(t\right)=2$ indicates that node $N_{i}$ fails at time t due to hardware/software failure events, and $\mathrm{Pr}\left(S_{N_{i}}\left(t\right)=2\right)$ represents the corresponding probability; $\lambda_{i}$ is the failure rate parameter in the exponential distribution; t≥0 represents the working time of the node. Let $S_{N_{i}}\left(t\right)=2$ be denoted as event $E_{N_{i}}^{2}\left(t\right)$, then the complementary event ${\bar{E}}_{N_{i}}^{2}\left(t\right)$ represents that the hardware/software of the node $N_{i}$ is working normally at time t. The following equations can be derived:

(2) $$\left\{\begin{array}{l} \mathrm{Pr}\left(E_{N_{i}}^{2}\left(t\right)\right)=\mathrm{Pr}\left(S_{N_{i}}\left(t\right)=2\right)=1-e^{-\lambda_{i}t}\\ \mathrm{Pr}\left({\bar{E}}_{N_{i}}^{2}\left(t\right)\right)=1-\mathrm{Pr}\left(E_{N_{i}}^{2}\left(t\right)\right)=e^{-\lambda_{i}t} \end{array}\right.$$

## 4. Analysis and Modeling of Node Energy Consumption Failure

Considering that there is no charging event for the batteries within VANETs nodes, and the node energy (battery capacity) is limited, when the remaining energy of the node is insufficient to support its normal operation, it is considered to be in an energy consumption failure state [37].

### 4.1. Node Energy Consumption Model

Let the remaining energy of node $N_{i}$ at time t be denoted by $E_{i}\left(t\right)$, and its initial energy be denoted by $E_{i}0$, i.e., $E_{i}\left(0\right)=E_{i}0$. The paper utilizes a first-order radio model to characterize the energy consumption process of VANETs nodes [34].

Let the energy consumed by node $N_{i}$ to send information $l_{T}^{N_{i}}\left(t\right)$ over a distance d at time t be denoted as $E_{T}^{N_{i}}\left(t\right)$, and the energy consumed by it to receive information $l_{R}^{N_{i}}\left(t\right)$ at time t be denoted as $E_{R}^{N_{i}}\left(t\right)$, where

(3) $$E_{T}^{N_{i}}\left(t\right)=l_{T}^{N_{i}}\left(t\right)\cdot \left(E_{elec}+\varepsilon_{amp}\cdot d^{\gamma_{i}\left(t\right)}\right),\;2\leq \gamma_{i}\left(t\right)\leq 4$$

(4) $$E_{R}^{N_{i}}\left(t\right)=l_{R}^{N_{i}}\left(t\right)\cdot E_{elec}$$

In the formula, $l_{T}^{N_{i}}\left(t\right)$ represents the amount of information sent by the node $N_{i}$ at time t, measured in bits; $E_{elec}$ represents the energy consumption of the circuit for transmitting 1 bit of information, $\varepsilon_{amp}$ is the power consumption coefficient of the power amplification circuit; $\gamma_{i}\left(t\right)$ represents the interference factor of the environment in which the node $N_{i}$ is located at time t; $l_{R}^{N_{i}}\left(t\right)$ represents the amount of information received by the $N_{i}$ at time t, measured in bits. The formula for calculating the remaining energy of node $N_{i}$ at a given moment t is:

(5) $$E_{i}\left(t\right)=E_{i}0-\int_{0}^{t}\left(E_{T}^{N_{i}}\left(t\right)+E_{R}^{N_{i}}\left(t\right)\right)dt$$

In the formula, $\int_{0}^{t}\left(E_{T}^{N_{i}}\left(t\right)+E_{R}^{N_{i}}\left(t\right)\right)dt$ represents the integral sum of energy consumed by node $N_{i}$ for sending and receiving information within the time interval.

When the remaining energy of a VANET node $N_{i}$ at time t is less than the energy threshold $E_{Th}$, the node $N_{i}$ experiences an energy depletion event, loses its communication function, and enters a failed state $S_{N_{i}}\left(t\right)=3$. In this context, $E_{Th}=\max\left(E_{T}^{N_{i}}\left(t\right),E_{R}^{N_{i}}\left(t\right)\right)$, if a VANET node loses either its information transmission or reception function, it is deemed to have experienced an energy consumption failure, and $S_{N_{i}}\left(t\right)=3\Leftrightarrow E_{i}\left(t\right)<E_{Th}$ an equivalent relationship holds. Denote $S_{N_{i}}\left(t\right)=3$ as event $E_{N_{i}}^{3}\left(t\right)$, where the complementary event indicates that the remaining energy of the node $N_{i}$ at time t is still sufficient to support the completion of its communication function. The probability calculation formula is:

(6) $$\begin{array}{l} \mathrm{Pr}\left({\bar{E}}_{N_{i}}^{3}\left(t\right)\right)=\mathrm{Pr}\left(E_{i}\left(t\right)\geq E_{Th}\right)\\ =\mathrm{Pr}\left(\left(E_{i}0-\int_{0}^{t}\left(E_{T}^{N_{i}}\left(t\right)+E_{R}^{N_{i}}\left(t\right)\right)dt\right)\geq E_{Th}\right) \end{array}$$

### 4.2. Analysis of the Amount of Information Processed by Nodes

**Figure 1:**
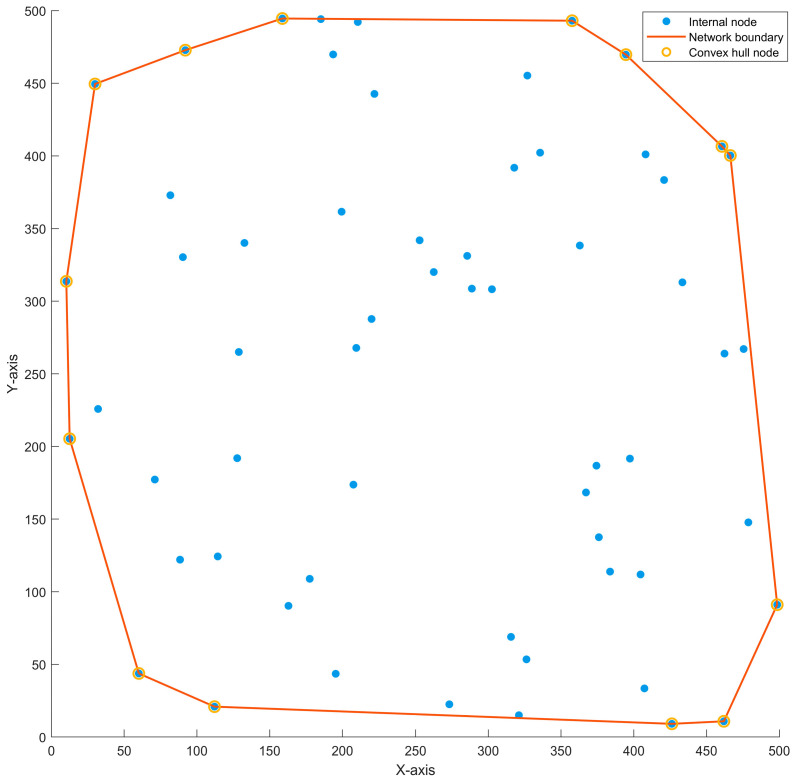
VANET and convex hull nodes.

Based on practical deployment scenarios, the information detected and sensed by the outermost nodes of VANETs during operation is often more valuable than that from internal nodes. For instance, it includes changes in terrain and topography ahead, obstructions caused by barriers, and dangerous sources such as epicenters or fire centers. According to computational geometry theory, the set of outermost nodes in VANETs constitutes what is known as the convex hull, as illustrated in Figure 1.

According to Figure 1, the internal nodes of VANETs are marked with blue solid dots, and the outermost nodes, namely the convex hull nodes, are marked with yellow circles. The lines connecting the convex hull nodes enclose the VANETs. The communication connection relationships between nodes are omitted in the figure. Considering the characteristics of VANETs, key control nodes $N^{C}$ do not appear at the location of convex hull nodes, so only general action nodes $N^{M}$ are judged on whether to move to the location of convex hull nodes. At time t, there are a total of $m_{1}$ key control nodes in VANETs, forming a set of control nodes $N^{C}=\left\{N_{i}^{C}|i=1,2,\ldots ,m_{1}\right\}$; a total of $m_{2}$ general action nodes move to the outermost, forming a set of convex hull nodes $N^{MCH}=\left\{N_{i}^{MCH}|i=1,2,\ldots ,m_{2}\right\}$; and another $m_{3}$ general action node is surrounded by the convex hull boundary, forming a set of general nodes $N^{MG}=\left\{N_{i}^{MG}|i=1,2,\ldots ,m_{3}\right\}$. Combining the VANETs graph theory model $G\left(V,E\right)$, we have:

(7) $$m_{1}+m_{2}+m_{3}=m$$

(8) $$V=N^{C}\cup N^{MCH}\cup N^{MG}=\left\{N_{1},N_{2},\ldots ,N_{m}\right\}$$

Considering that during the task process, the information transmission cycles of various types of nodes in VANETs are inconsistent with the requirements:

(1) For control nodes $N^{C}$, these nodes are responsible for sending command and control information $l_{C2}$ to all nodes within the network at the initial moment t=0. Based on the situational information collected by each convex hull node during the task process, they make response decisions and then send the updated control information $l_{C2}$ to the corresponding action nodes. For ease of analysis, the paper assumes that the period for the control nodes $N^{C}$ to send real-time command information is $T_{SC}$.

(2) For convex hull nodes $N^{MCH}$, this type of node needs to send situational awareness information $l_{SC}$ to the control node at regular intervals $T_{SC}$, and send coordinate information $l_{CO}$ to the control node at regular intervals $T_{CO}$.

(3) For general nodes $N^{MG}$, they need to forward (as relay nodes) situational information $l_{SC}$ from convex hull nodes and send coordinate information $l_{CO}$ to control nodes. The relay information depends on the timing of the convex hull node’s situational awareness information transmission, that is, the interval period $T_{SC}$, while the coordinate information needs to be sent every interval period $T_{CO}$. The above nodes and their types of information transmission and reception are shown in Table 2.

Based on Table 2 and the aforementioned analysis, the paper considers three types of information transmission situations (ITS) within VANETs, as shown in Table 3.

In ITS1, the node $N^{C}$ sends control information to all nodes in VANETs, unifying node actions and ensuring that the overall network always aims to complete the predetermined task. The timing of its transmission includes both the initial moment t=0 and the moment after receiving situational information from the convex hull node $N^{MCH}$ and making decisions: $0,\;T_{SC},\;2T_{SC},\;3T_{SC},\;\ldots$ Furthermore, in ITS1, any relay node is only responsible for receiving control information from the source node $N^{C}$ and forwarding it to the sink node $N^{MCH}$, without generating additional self-coordinate information $l_{CO}$.

In ITS2, as the convex hull nodes $N^{MCH}$ move to the outermost perimeter of VANETs, they need to send situational awareness information $l_{SC}$ to the nodes $N^{C}$ to assist them in making decisions for the next action. The timing of this transmission is $0,\;T_{SC},\;2T_{SC},\;3T_{SC},\;\ldots$ Furthermore, in ITS2, any relay node is only responsible for receiving situational awareness information from the source node $N^{MCH}$ and forwarding it to the sink node $N^{C}$, without generating additional self-coordinate information $l_{CO}$.

In ITS3, all working nodes within VANETs are required to send their own coordinate information $l_{CO}$ to the node $N^{C}$ to ensure that the control node is aware of the survival and distribution of network nodes. The timing for sending this information is $0,\;T_{CO},\;2T_{CO},\;3T_{CO},\;\ldots$. Additionally, in ITS3, relay nodes are not only responsible for receiving coordinate information from source nodes and predecessor nodes, but also generate their own coordinate information. After all information is aggregated, it is then sent to successor nodes. That is, on the same routing path, relay nodes closer to the sink node $N^{C}$ have a greater amount of information to process than those farther away from the sink node.

**Figure 2:**
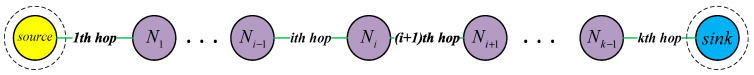
k-hop connected path.

Consider a k-hop connected path consisting of k+1 nodes, where the source node $N_{source}$ is marked in yellow and the sink node $N_{sink}$ is marked in blue. The k−1 intermediate nodes $N_{i}\left(i=1,2,\ldots ,k-1\right)$ serve as relay nodes, facilitating communication connectivity between the source and sink nodes through single-hop links, as illustrated in Figure 2.

Without considering communication delay, the amount of information $l_{T}^{N_{source}}$ sent by the source node during the establishment of a k-hop connected path is first received and stored by the node $N_{1}$. The information sent by the node $N_{1}$ to $N_{2}$ consists of two parts: the amount of information received from the source node and requiring relaying, $l_{R}^{N_{1}}=l_{T}^{N_{source}}$, and the information generated by the node $N_{1}$ and intended for the sink node, $l_{G}^{N_{1}}$. That is, $l_{T}^{N_{1}}=l_{T}^{N_{source}}+l_{G}^{N_{1}}$. For the node $N_{2}$, the amount of information it receives is equal to the amount of information sent by the preceding node $N_{1}$, and the information it sends consists of two parts: the amount of information received from the preceding node and requiring relaying, $l_{R}^{N_{2}}=l_{T}^{N_{1}}$, and the information generated by the node and needing to be sent to the subsequent node $N_{3}$ in the routing path. That is, $l_{T}^{N_{2}}=l_{R}^{N_{2}}+l_{G}^{N_{2}}=l_{T}^{N_{1}}+l_{G}^{N_{2}}=l_{T}^{N_{source}}+l_{G}^{N_{1}}+l_{G}^{N_{2}}$.

By analogy, in this path, the ith node $N_{i}$ forms the ith hop with node $N_{i-1}$ and the $\left(i+1\right)$th hop with node $N_{i+1}$. The amount of information received by node $N_{i}$ is equal to the amount of information sent by the preceding node $N_{i-1}$ and has $l_{R}^{N_{i}}=l_{T}^{N_{i-1}}$. The information sent by node $N_{i}$ to $N_{i+1}$ consists of two parts: the amount of information received from the preceding node $N_{i-1}$ and needs to be relayed $l_{R}^{N_{i}}=l_{T}^{N_{i-1}}$, and the amount of information generated by node $N_{i}$ and needs to be sent to the sink node $l_{G}^{N_{i}}$. Therefore, in a route consisting of a k-hop connected path, the mathematical expression of the amount of information that the relay node $N_{i}$ at ith hop needs to process is:

(9) $$l_{R}^{N_{i}}=l_{T}^{N_{i-1}}=l_{T}^{N_{source}}+\sum_{a=1}^{i-1}l_{G}^{N_{a}}$$

(10) $$l_{T}^{N_{i}}=l_{T}^{N_{i-1}}+l_{G}^{N_{i}}=l_{T}^{N_{source}}+\sum_{a=1}^{i}l_{G}^{N_{a}}$$

(11) $$l_{T}^{N_{i}}-l_{R}^{N_{i}}=l_{G}^{N_{i}}$$

**Figure 3:**
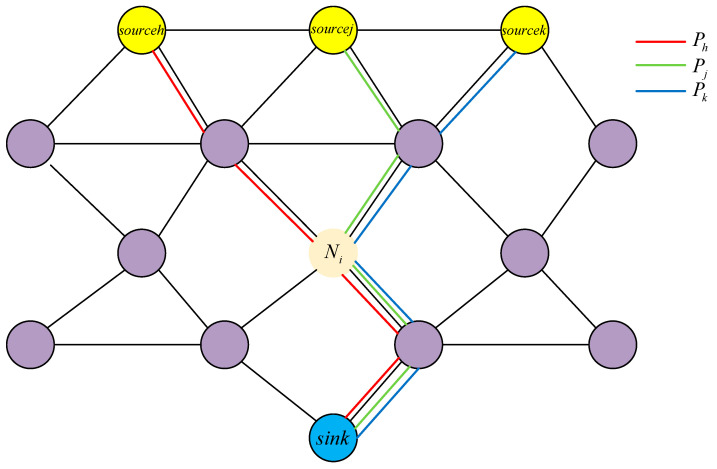
A single node belonging to multiple routing paths simultaneously.

Furthermore, there may be Q multiple routing paths $P=\left\{P_{1},P_{2},\ldots ,P_{Q}\right\}$ within VANETs, and nodes $N_{i}$ may act as relay nodes to send and receive information across multiple routing paths, as shown in Figure 3. The amount of information $l_{R}^{N_{i}}$ and $l_{T}^{N_{i}}$ processed at this time are as follows:

(12) $$l_{R}^{N_{i}}=\sum_{v=1}^{Q}\zeta_{N_{i}}^{P_{v}R}l_{R}^{P_{v}N_{i}}=\sum_{v=1}^{Q}\zeta_{N_{i}}^{P_{v}R}\left(l_{T}^{sourceP_{v}}+\sum_{a=1}^{InP_{v}N_{i}-1}\zeta_{N_{a}}^{P_{v}T}l_{G}^{N_{a}}\right)$$

(13) $$l_{T}^{N_{i}}=l_{R}^{N_{i}}+l_{G}^{N_{i}}=\sum_{v=1}^{Q}\zeta_{N_{i}}^{P_{v}R}\left(l_{T}^{sourceP_{v}}+\sum_{a=1}^{InP_{v}N_{i}-1}\zeta_{N_{a}}^{P_{v}T}l_{G}^{N_{a}}\right)+l_{G}^{N_{i}}$$

In the formula, Q represents the number of routing paths existing within VANETs; $P_{v}\left(v=1,2,\ldots ,Q\right)$ denotes the vth routing path; $\zeta_{N_{i}}^{P_{v}R}$ is a binary variable, $\zeta_{N_{i}}^{P_{v}R}=1$ indicating node $N_{i}$ receives information in the routing path $P_{v}$, otherwise $\zeta_{N_{i}}^{P_{v}R}=0$; $l_{R}^{P_{v}N_{i}}$ represents the amount of information received by the node $N_{i}$ in the routing path $P_{v}$; $l_{T}^{sourceP_{v}}$ denotes the amount of information sent by the source node $sourceP_{v}$ in the routing path $P_{v}$; $InP_{v}N_{i}$ is the index value of the hop count where the node $N_{i}$ is located in the routing path $P_{v}$; $InP_{v}N_{i}-1$ denotes the number of predecessor nodes of the node $N_{i}$ in the routing path $P_{v}$; $N_{a}\left(a=1,2,\ldots ,InP_{v}N_{i}-1\right)$ represents the ath predecessor node of the node $N_{i}$ in the routing path $P_{v}$; $\zeta_{N_{a}}^{P_{v}T}$ is a binary variable, $\zeta_{N_{a}}^{P_{v}T}=1$ indicating node $N_{a}$ sends information generated by itself in the routing path $P_{v}$, otherwise $\zeta_{N_{a}}^{P_{v}T}=0$; $\sum_{a=1}^{InP_{v}N_{i}-1}\zeta_{N_{a}}^{P_{v}T}l_{G}^{N_{a}}$ represents the sum of the amount of information generated by predecessor nodes and transmitted in the routing path $P_{v}$ for the node $N_{i}$; $\sum_{v=1}^{Q}\zeta_{N_{i}}^{P_{v}R}l_{R}^{P_{v}N_{i}}$ denotes traversing all routing paths within VANETs and summing the amount of information received by the node $N_{i}$ in each routing path.

Based on Table 3, the information processing volume $l_{R}^{N_{i}}$ and $l_{T}^{N_{i}}$ of relay nodes $N_{i}$ in VANETs under 3 information transmission situations can be obtained:

ITS1 node processing information volume:

(14) $$l_{R}^{N_{i}}\left(t\right)=\sum_{v=1}^{Q}\zeta_{N_{i}}^{P_{v}R}l_{R}^{P_{v}N_{i}}\left(t\right)=\sum_{v=1}^{Q}\zeta_{N_{i}}^{P_{v}R}\left(l_{T}^{sourceP_{v}}\left(t\right)+\sum_{a=1}^{InP_{v}N_{i}-1}\zeta_{N_{a}}^{P_{v}T}l_{G}^{N_{a}}\left(t\right)\right)=\sum_{v=1}^{Q}\zeta_{N_{i}}^{P_{v}R}l_{C2}$$

(15) $$l_{T}^{N_{i}}\left(t\right)=l_{R}^{N_{i}}+l_{G}^{N_{i}}\left(t\right)=\sum_{v=1}^{Q}\zeta_{N_{i}}^{P_{v}R}\left(l_{T}^{sourceP_{v}}\left(t\right)+\sum_{a=1}^{InP_{v}N_{i}-1}\zeta_{N_{a}}^{P_{v}T}l_{G}^{N_{a}}\left(t\right)\right)+0=\sum_{v=1}^{Q}\zeta_{N_{i}}^{P_{v}R}l_{C2}$$

(16) $$l_{G}^{N_{i}}\left(t\right)=0$$

ITS2 node processing information volume:

(17) $$l_{R}^{N_{i}}\left(t\right)=\sum_{v=1}^{Q}\zeta_{N_{i}}^{P_{v}}l_{R}^{P_{v}N_{i}}\left(t\right)=\sum_{v=1}^{Q}\zeta_{N_{i}}^{P_{v}}\left(l_{T}^{sourceP_{v}}+\sum_{a=1}^{InP_{v}N_{i}-1}\zeta_{N_{a}}^{P_{v}T}l_{G}^{N_{a}}\left(t\right)\right)=\sum_{v=1}^{Q}\zeta_{N_{i}}^{P_{v}}l_{SC}$$

(18) $$l_{T}^{N_{i}}\left(t\right)=l_{R}^{N_{i}}\left(t\right)+l_{G}^{N_{i}}\left(t\right)=\sum_{v=1}^{Q}\zeta_{N_{i}}^{P_{v}}\left(l_{T}^{sourceP_{v}}+\sum_{a=1}^{InP_{v}N_{i}-1}\zeta_{N_{a}}^{P_{v}T}l_{G}^{N_{a}}\left(t\right)\right)+0=\sum_{v=1}^{Q}\zeta_{N_{i}}^{P_{v}}l_{SC}$$

(19) $$l_{G}^{N_{i}}\left(t\right)=0$$

ITS3 node processing information volume:

(20) $$\begin{array}{l} l_{R}^{N_{i}}\left(t\right)=\sum_{v=1}^{Q}\zeta_{N_{i}}^{P_{v}}l_{R}^{P_{v}N_{i}}\left(t\right)=\sum_{v=1}^{Q}\zeta_{N_{i}}^{P_{v}}\left(l_{T}^{sourceP_{v}}\left(t\right)+\sum_{a=1}^{InP_{v}N_{i}-1}\zeta_{N_{a}}^{P_{v}T}l_{G}^{N_{a}}\left(t\right)\right)\\ =\sum_{v=1}^{Q}\zeta_{N_{i}}^{P_{v}}\left(l_{CO}+\sum_{a=1}^{InP_{v}N_{i}-1}\zeta_{N_{a}}^{P_{v}T}l_{CO}\right) \end{array}$$

(21) $$\begin{array}{l} l_{T}^{N_{i}}\left(t\right)=l_{R}^{N_{i}}\left(t\right)+l_{G}^{N_{i}}\left(t\right)=\sum_{v=1}^{Q}\zeta_{N_{i}}^{P_{v}}\left(l_{T}^{sourceP_{v}}\left(t\right)+\sum_{a=1}^{InP_{v}N_{i}-1}\zeta_{N_{a}}^{P_{v}T}l_{G}^{N_{a}}\left(t\right)\right)+l_{CO}\\ =\sum_{v=1}^{Q}\zeta_{N_{i}}^{P_{v}}\left(l_{CO}+\sum_{a=1}^{InP_{v}N_{i}-1}\zeta_{N_{a}}^{P_{v}T}l_{CO}\right)+l_{CO} \end{array}$$

(22) $$l_{G}^{N_{i}}\left(t\right)=l_{CO}$$

At a given moment t, multiple information transmission situations may coexist within VANETs, such as: if $\mathrm{mod}\left(t,T_{SC}\times T_{CO}\right)=0$, there were simultaneous ITS1, ITS2, and ITS3 within VANETs. If $\mathrm{mod}\left(t,T_{SC}\right)=0$, there were simultaneous ITS1 and ITS2 within VANETs; if $\mathrm{mod}\left(t,T_{CO}\right)=0$, there was only ITS3 within VANETs. If a node $N_{i}$ belongs to multiple information transmission situations and multiple routing paths simultaneously at a given moment, its information processing volume should be accumulated not only for multiple information transmission situations but also for multiple routing paths. Here, $\mathrm{mod}\left(a,b\right)$ is the remainder of a to b.

### 4.3. Routing Path Recognition Algorithm

Based on the aforementioned analysis, there are primarily 3 types of information transmission situations within VANETs, each with distinct information flow rules that require sequential analysis. To prevent excessive energy consumption of VANETs nodes due to repeated information transmission, this paper proposes corresponding routing path identification algorithms and pseudocodes for each of the 3 information transmission situations.

ITS1 routing path identification algorithm

At the moment $t=0,T_{SC},2T_{SC},\ldots$, the control node $N^{C}$ in VANETs sends command and control information as well as task information to all remaining nodes ($N^{MCH}\cup N^{MG}$). During this process, relay nodes only forward the information and do not generate their own coordinate information, ensuring the conservation of information flow within VANETs. The routing path identification algorithm at this time is as follows:

(1) Generate VANETs links information: Based on coordinate information $CoN_{i}\left(t\right)\left(N_{i}\in V;t=0,T_{SC},2T_{SC},\ldots \right)$, generate a Euclidean distance matrix between nodes for all normally operating nodes $N_{i}$ within VANETs. Further generate a time-varying binary communication connectivity adjacency matrix $A_{B}\left(t\right)$ through logical operations $A_{B}\left(t\right)=M_{d}\left(t\right)\leq d_{Th}$. Obtain all link information through the matrix $A_{B}\left(t\right)$ and its constituent elements.

(2) Classify VANETs nodes: Determine the control node $N^{C}$ within VANETs based on the command hierarchy (assuming there is only one control node), and determine the convex hull node set $N^{MCH}=\left\{N_{i}^{MCH}|i=1,2,\ldots ,m_{2}\right\}$ and general node set $N^{MG}=\left\{N_{i}^{MG}|i=1,2,\ldots ,m_{3}\right\}$ based on coordinate location information, where $1+m_{2}+m_{3}=m$. At this point, the set of all nodes waiting to receive task information is $V_{ToR}=N^{MCH}\cup N^{MG}$, the set of nodes that have received task information is $V_{Rd}=\emptyset$, and there are $V=\left\{N^{C}\right\}\cup V_{ToR}\cup V_{Rd}=\left\{N^{C}\right\}\cup N^{MCH}\cup N^{MG}$.

(3) Determine the routing path for transmitting task information to the current convex hull set nodes $N^{MCH}$: Use the control node $N^{C}$ as the source node, and all convex hull nodes $N_{i}^{MCH}$($i=1,2,\ldots ,m_{2}$) as the sink node set $V_{sink}=N^{MCH}$. Identify the minimum path set between the source node and each sink node $N_{i}^{MCH}$ in turn, and determine the routing path $P_{C-MCH_{i}}$ between each pair of nodes $\left(N^{C},N_{i}^{MCH}\right)$ according to the shortest path routing rule.

(4) Identify all received task information nodes: Let the set of relay nodes involved in the routing path $P_{C-MCH_{i}}$ between node pairs $\left(N^{C},N_{i}^{MCH}\right)$ be $V_{C-MCH_{i}}^{relay}=\left\{N_{C-MCH_{i}}^{re1},N_{C-MCH_{i}}^{re2},\ldots \right\}$, then the nodes in the routing path $P_{C-MCH_{i}}$ that have received task information are $\left\{N_{i}^{MCH}\right\}\cup V_{C-MCH_{i}}^{relay}$, update $V_{Rd}=V_{Rd}\cup \left(\left\{N_{i}^{MCH}\right\}\cup V_{C-MCH_{i}}^{relay}\right)$, update $V_{ToR}=V_{ToR}\backslash \left(\left\{N_{i}^{MCH}\right\}\cup V_{C-MCH_{i}}^{relay}\right)$. After traversing all the routing paths in step (3), update $V_{Rd}$ and $V_{ToR}$.

(5) Establish new convex hull set nodes: In the updated set of task information nodes to be received, determine the convex hull nodes based on the coordinate position information. If the updated convex hull node set $N^{MCH}\neq \emptyset$, proceed to step (3); otherwise, proceed to step (6).

(6) Identify all routing paths: Collect all routing paths that appear in the above steps to obtain a complete set of routing paths $P_{C-N}$ for transmitting VANETs task information to avoid duplicate sending.

The pseudocode of the ITS1 routing path identification Algorithm 1 is as follows.
**Algorithm 1.** ITS1 routing path identification algorithm.Input:Source node $N^{C}$; Sink node set $V_{sink}=N^{MCH}\cup N^{MG}$; Node coordinates $CoN_{i}\left(t\right)$; communication distance threshold $d_{Th}$
Output:Complete routing path set $P_{C-N}$
1.calculate $M_{d}\left(t\right)$
2.generate $A_{B}\left(t\right)=M_{d}\left(t\right)\leq d_{Th}$
3.// Generate link information4.classify $N^{C}$, $N^{MCH}$, $N^{MG}$
5.set $V_{ToR}=N^{MCH}\cup N^{MG}$
6.set $V_{Rd}=\emptyset$
7.// Initialize node sets8.While $N^{MCH}\neq \emptyset$
9.// Find paths from $N^{C}$ to $N^{MCH}$
10.  For $N_{i}^{MCH}\in N^{MCH}$
11.     find $P_{C-MCH_{i}}$
12.     extract $V_{C-MCH_{i}}^{relay}$
13.     update $V_{Rd}=V_{Rd}\cup \left\{N_{i}^{MCH}\right\}\cup V_{C-MCH_{i}}^{relay}$
14.     update $V_{ToR}=V_{ToR}\backslash \left(\left\{N_{i}^{MCH}\right\}\cup V_{C-MCH_{i}}^{relay}\right)$
15.     update $P_{C-N}=P_{C-N}\cup P_{C-MCH_{i}}$
16.  End For17.  re-determine $N^{MCH}$
18.End While19.Output $P_{C-N}$


ITS2 routing path identification algorithm

At the moment $t=0,T_{SC},2T_{SC},\ldots$, all convex hull nodes in VANETs send situation information to the control node $N^{C}$. During this process, relay nodes only forward the information and do not generate their own coordinate information, ensuring the conservation of information flow within VANETs. The routing path identification algorithm is as follows:

(1) Generate VANETs links information: Based on coordinate information $CoN_{i}\left(t\right)\left(N_{i}\in V;t=0,T_{SC},2T_{SC},\ldots \right)$, generate a Euclidean distance matrix between nodes for all normally operating nodes $N_{i}$ within VANETs. Further generate a time-varying binary communication connectivity adjacency matrix $A_{B}\left(t\right)$ through logical operations $A_{B}\left(t\right)=M_{d}\left(t\right)\leq d_{Th}$. Obtain all link information through the matrix $A_{B}\left(t\right)$ and its constituent elements.

(2) Classify VANETs nodes: Determine the control nodes $N^{C}$ within VANETs based on command hierarchy, and determine the convex hull node set $N^{MCH}=\left\{N_{i}^{MCH}|i=1,2,\ldots ,m_{2}\right\}$ and general node set $N^{MG}=\left\{N_{i}^{MG}|i=1,2,\ldots ,m_{3}\right\}$ based on coordinate location information. At this point, the nodes that waiting to receive situational information are only the control nodes $N^{C}$, i.e., $V_{ToR}=\left\{N^{C}\right\}$.

(3) Determine the transmission routing path for the situation information from the convex hull set nodes $N^{MCH}$ to the control node $N^{C}$: Form a source node set $V_{source}=N^{MCH}$ consisting of all convex hull nodes $N_{i}^{MCH}$($i=1,2,\ldots ,m_{2}$), identify the minimum path set between the source nodes and the sink node in turn, and determine the routing path $P_{MCH_{i}-C}$ between each pair of nodes $\left(N_{i}^{MCH},N^{C}\right)$ according to the shortest path routing rule.

(4) Identify all routing paths: Collect all routing paths that appear in the above steps to obtain a complete routing paths set $P_{N-C}$ for transmitting VANETs situational information to avoid duplicate transmissions.

The pseudocode of the ITS2 routing path identification Algorithm 2 is as follows.
**Algorithm 2.** ITS2 routing path identification algorithm.Input:Source node set $V_{source}=N^{MCH}$; Sink node $N^{C}$; Node coordinates $CoN_{i}\left(t\right)$; communication distance threshold $d_{Th}$
Output:Complete routing path set $P_{N-C}$
1.calculate $M_{d}\left(t\right)$
2.generate $A_{B}\left(t\right)=M_{d}\left(t\right)\leq d_{Th}$
3.// Generate link information4.For $N_{i}^{MCH}\in V_{source}$:5.// Find paths from $N^{MCH}$ to $N^{C}$
6.  find $P_{MCH_{i}-C}$
7.  update $P_{N-C}=P_{N-C}\cup P_{MCH_{i}-C}$
8.End For9.Output $P_{N-C}$


ITS3 routing path identification algorithm

At any given moment $t=0,T_{CO},2T_{CO},\ldots$, all working nodes in VANETs must transmit their own coordinate information to the control node $N^{C}$. During this process, relay nodes not only need to receive coordinate information from the source node and the preceding node, but also generate their own coordinate information. Once all information is collected and processed, it will continue to be transmitted to the subsequent nodes. This means that the information flow within VANETs is non-conservative and increases during the transmission process from the source node to the sink node. The routing path identification algorithm in this case is as follows:

(1) Generate VANETs links information: Based on coordinate information $CoN_{i}\left(t\right)\left(N_{i}\in V;t=0,T_{SC},2T_{SC},\ldots \right)$, generate a Euclidean distance matrix between nodes for all normally operating nodes $N_{i}$ within VANETs. Further generate a time-varying binary communication connectivity adjacency matrix $A_{B}\left(t\right)$ through logical operations $A_{B}\left(t\right)=M_{d}\left(t\right)\leq d_{Th}$. Obtain all link information through the matrix $A_{B}\left(t\right)$ and its constituent elements.

(2) Classify VANETs nodes: Determine the control nodes $N^{C}$ within VANETs based on the command hierarchy, and identify the convex hull node set $N^{MCH}=\left\{N_{i}^{MCH}|i=1,2,\ldots ,m_{2}\right\}$ and general node set $N^{MG}=\left\{N_{i}^{MG}|i=1,2,\ldots ,m_{3}\right\}$ based on coordinate location information. At this point, the set of nodes with coordinate information to be sent is $V_{ToS}=N^{MCH}\cup N^{MG}$, and the set of nodes with coordinate information already sent is $V_{Sd}=\emptyset$. There is only one sink node $N^{C}$, and it has $V=\left\{N^{C}\right\}\cup V_{ToS}\cup V_{Sd}=\left\{N^{C}\right\}\cup N^{MCH}\cup N^{MG}$.

(3) Determine the transmission routing path for coordinate information of the current convex hull node set $N^{MCH}$: Form a source node set $V_{source}=N^{MCH}$ consisting of all convex hull nodes $N_{i}^{MCH}$($i=1,2,\ldots ,m_{2}$), identify the minimum path set between the source nodes and the sink node $N^{C}$ in turn, and determine the routing path $P_{MCH_{i}-C}$ between each pair of nodes $\left(N_{i}^{MCH},N^{C}\right)$ according to the shortest path routing rule.

(4) Identify all nodes that have sent coordinate information: Let the set of relay nodes involved in the routing path $P_{MCH_{i}-C}$ between node pairs $\left(N_{i}^{MCH},N^{C}\right)$ be denoted by $V_{MCH_{i}-C}^{relay}=\left\{N_{MCH_{i}-C}^{re1},N_{MCH_{i}-C}^{re2},\ldots \right\}$, then the nodes that have sent coordinate information in the routing path $P_{MCH_{i}-C}$ are $\left\{N_{i}^{MCH}\right\}\cup V_{MCH_{i}-C}^{relay}$, update $V_{Sd}=V_{Sd}\cup \left(\left\{N_{i}^{MCH}\right\}\cup V_{MCH_{i}-C}^{relay}\right)$, update $V_{ToS}=V_{ToS}\backslash \left(\left\{N_{i}^{MCH}\right\}\cup V_{MCH_{i}-C}^{relay}\right)$. After traversing all the routing paths in step (3), update $V_{Sd}$ and $V_{ToS}$.

(5) Establish new convex hull set nodes: In the updated set $V_{ToS}$ of nodes with coordinate information to be sent, determine the convex hull nodes based on the coordinate position information. If the convex hull node set $N^{MCH}\neq \emptyset$, proceed to step (3); otherwise, proceed to step (6).

(6) Identify all routing paths: Collect all routing paths that appeared in the above steps to obtain the set $P_{N-C}$ of all routing paths for transmitting VANETs coordinate information while avoiding duplicate transmissions.

The pseudocode of the ITS3 routing path identification Algorithm 3 is as follows.
**Algorithm 3.** ITS3 routing path identification algorithm.Input:Source node set $V_{source}=N^{MCH}\cup N^{MG}$; Sink node $N^{C}$; Node coordinates $CoN_{i}\left(t\right)$; communication distance threshold $d_{Th}$
Output:Complete routing path set $P_{N-C}$
1.calculate $M_{d}\left(t\right)$
2.generate $A_{B}\left(t\right)=M_{d}\left(t\right)\leq d_{Th}$
3.// Generate link information4.classify $N^{C}$, $N^{MCH}$, $N^{MG}$
5.set $V_{ToS}=N^{MCH}\cup N^{MG}$
6.set $V_{Sd}=\emptyset$
7.// Initialize node sets8.While $N^{MCH}\neq \emptyset$
9.// Find paths from $N^{MCH}$ to $N^{C}$
10.  For $N_{i}^{MCH}\in N^{MCH}$
11.     find $P_{MCH_{i}-C}$
12.     extract $V_{MCH_{i}-C}^{relay}$
13.     update $V_{Sd}=V_{Sd}\cup \left(\left\{N_{i}^{MCH}\right\}\cup V_{MCH_{i}-C}^{relay}\right)$
14.     update $V_{ToS}=V_{ToS}\backslash \left(\left\{N_{i}^{MCH}\right\}\cup V_{MCH_{i}-C}^{relay}\right)$
15.     update $P_{N-C}=P_{N-C}\cup P_{MCH_{i}-C}$
16.  End For17.  re-determine $N^{MCH}$
18.End While19.Output $P_{N-C}$


To evaluate the computational efficiency of the proposed routing path recognition algorithm (applicable to ITS1, ITS2, and ITS3 scenarios with consistent logical steps), its time complexity is analyzed based on the node scale m (total number of nodes in VANETs) and edge scale n (number of links).

(1) Core Steps and Time Complexity Breakdown

The algorithm consists of five key steps, with time complexity dominated by shortest path search and link information generation.

Generation of Link Information. First, the Euclidean distance matrix $M_{d}\left(t\right)$ is computed using node coordinates, involving $\frac{m\left(m-1\right)}{2}$ node pair distance calculations with time complexity $O\left(m^{2}\right)$. Then, a binary adjacency matrix $A_{B}\left(t\right)$ is generated via logical operations $d_{ij}\leq d_{Th}$, which requires traversing $m^{2}$ elements of $M_{d}\left(t\right)$, with time complexity $O\left(m^{2}\right)$. Total for this step: $O\left(m^{2}\right)$.

Node Classification. Identification of the key control node $N^{C}$: Directly determined by command hierarchy (assumed 1 control node), time complexity $O\left(1\right)$. Convex hull node $N^{MCH}$ calculation: Implemented using the Andrew’s monotone chain algorithm (efficient for 2D convex hulls), with time complexity $O\left(m\log m\right)$. General node $N^{MG}$ classification: Derived via set difference $V-N^{C}-N^{MCH}$, with time complexity $O\left(m\right)$. Total for this step: $O\left(m\log m\right)$.

Shortest Path Search. The algorithm adopts the Breadth-First Search (BFS) for shortest path routing. Source node count S and destination node count D: Both are $O\left(m\right)$ (e.g., ITS1: $S=1\left(N^{C}\right)$, $D=O\left(m\right)\left(N^{MCH}\right)$; ITS2/ITS3: $S=O\left(m\right)\left(N^{MCH}\right)$, $D=1\left(N^{C}\right)$). Time per BFS: $E=O\left(m^{2}\right)$, BFS traverses all nodes and edges, time $O\left(m+n\right)=O\left(m^{2}\right)$. Total for this step: $O\left(S\times D\times \left(m+n\right)\right)=O\left(m\times m^{2}\right)=O\left(m^{3}\right)$.

Node Set Update. Nodes that have received/transmitted information are added to $V_{Rd}/V_{Sd}$ and removed from $V_{ToR}/V_{ToS}$. This involves traversing $O\left(m\right)$ routing paths, each with up to $O\left(m\right)$ nodes. Total for this step: $O\left(m^{2}\right)$.

Path Deduplication and Collection. Duplicate paths are removed by comparing $O\left(m\right)$ paths (each of length $O\left(m\right)$) using a hash set. Total for this step: $O\left(m^{2}\right)$.

(2) Overall Time Complexity

The overall time complexity is determined by the dominant step (shortest path search): $O\left(m^{3}\right)$.

## 5. Node Mobility Analysis and Communication Link Modeling

### 5.1. Node Position Update Equation

The paper assumes that VANETs are deployed on a two-dimensional plane to perform tasks, where any node $N_{i}$$\left(i=1,2,\ldots ,m\right)$ is positioned by plane coordinates $CoN_{i}=\left(x_{i},y_{i}\right)$. It is stipulated that the speed of each node $N_{i}$ falls within the interval $\left[v_{\min},v_{\max}\right]$, and the orientation vectors take values $O_{i}=\left(\cos\theta_{i},\sin\theta_{i}\right)$, while $\theta_{i}$ falls within a certain interval $\left[0,2\pi \right]$. For any node $N_{i}$, its position update equation at time t+Δt can be expressed as:

(23) $$\left\{\begin{array}{l} x_{i}\left(t+\Delta t\right)=x_{i}\left(t\right)+\Delta t\cdot v_{i}\left(t\right)\cdot \cos\theta_{i}\left(t+\Delta t\right)\\ y_{i}\left(t+\Delta t\right)=y_{i}\left(t\right)+\Delta t\cdot v_{i}\left(t\right)\cdot \sin\theta_{i}\left(t+\Delta t\right)\\ v_{i}\left(t+\Delta t\right)=v_{\min}+r\cdot \left(v_{\max}-v_{\min}\right) \end{array}\right.$$

In the formula, $x_{i}\left(t+\Delta t\right)$ and $y_{i}\left(t+\Delta t\right)$ represent the horizontal and vertical coordinates of the node $N_{i}$ at time t+Δt, respectively; $v_{i}\left(t\right)\cdot \cos\theta_{i}\left(t\right)$ and $v_{i}\left(t\right)\cdot \sin\theta_{i}\left(t\right)$ represent the components of velocity $v_{i}\left(t\right)$ in the x and y axes at time t, respectively; r is a random number in the interval $\left[0,1\right]$. That is, the position of the node $N_{i}$ at time t+Δt depends on its position $CoN_{i}\left(t\right)$, velocity $v_{i}\left(t\right)$ at time t, displacement in the time increment Δt, and orientation $O_{i}\left(t+\Delta t\right)$ at time t+Δt.

For a node $N_{i}$, let its initial coordinate be $CoN_{i}\left(0\right)=\left(x_{i}\left(0\right),y_{i}\left(0\right)\right)$. After considering the location update formula, its coordinate at time t can be solved through the formula:

(24) $$\left\{\begin{array}{l} x_{i}\left(t\right)=x_{i}\left(0\right)+\int_{0}^{t}v_{i}\left(t\right)\cdot \cos\theta_{i}\left(t+\Delta t\right)dt\approx x_{i}\left(0\right)+\sum_{\Delta t\to 0}\Delta t\cdot v_{i}\left(\Delta t\right)\cdot \cos\theta_{i}\left(\Delta t\right)\\ y_{i}\left(t\right)=y_{i}\left(0\right)+\int_{0}^{t}v_{i}\left(t\right)\cdot \sin\theta_{i}\left(t+\Delta t\right)dt\approx y_{i}\left(0\right)+\sum_{\Delta t\to 0}\Delta t\cdot v_{i}\left(\Delta t\right)\cdot \sin\theta_{i}\left(\Delta t\right) \end{array}\right.$$

The Euclidean distance between two nodes $N_{i},\;N_{j}\left(i\neq j\right)$ at a given time t can be expressed as:

(25) $$d_{ij}\left(t\right)=\sqrt{{\left(x_{i}\left(t\right)-x_{j}\left(t\right)\right)}^{2}+{\left(y_{i}\left(t\right)-y_{j}\left(t\right)\right)}^{2}}$$

By traversing all node pairs within VANETs, a Euclidean distance matrix between nodes can be generated:

(26) $$M_{d}\left(t\right)=\left[\begin{array}{cccc} d_{11}\left(t\right) & d_{12}\left(t\right) & \ldots & d_{1m}\left(t\right)\\ d_{21}\left(t\right) & d_{22}\left(t\right) & \ldots & d_{2m}\left(t\right)\\ \vdots & \vdots & \ddots & \vdots \\ d_{m1}\left(t\right) & d_{m2}\left(t\right) & \ldots & d_{mm}\left(t\right) \end{array}\right]$$

Any element in matrix $M_{d}\left(t\right)$ represents the Euclidean distance $d_{ij}\left(t\right)$ between nodes $N_{i}$ and $N_{j}$ $\left(i\neq j\right)$ at a given moment t. By symmetry, $d_{ij}\left(t\right)=d_{ji}\left(t\right)$ for i≠j and $d_{ii}\left(t\right)=0$ (i.e., self-loops are excluded).

### 5.2. Couzin-Leader Improved Model

In practical applications, nodes of self-organizing networks (represented by VANETs nodes) exhibit a high degree of organization and discipline during task execution, and cooperate with each other to achieve common goals. At this time, the random nature of node movement is relatively weakened, and the overall behavior of the network system becomes more prominent. However, existing research often assumes that node movement follows benchmark models such as the Random Waypoint Mobility Model (RWPM) and the Random Direction Mobility Model (RDMM), which emphasize individual behavior in simulation processes and under-characterize the overall movement characteristics of the network [35]. Considering the special control relationships that exist in VANETs, this paper introduces the Couzin-leader mobility model [5] to provide a more realistic description of both arbitrary node movement and overall network behavior. The Couzin-leader model is an improvement of the Couzin model [38] and has certain advantages in depicting self-organizing behavior. It has been widely applied in communication networks such as drone swarms [6,22]. It mainly includes two key roles and four motion rules.

The two key roles in group movement are the leader and the follower. As the key minority in the network, the leader has the highest level of understanding of the overall task information of the network and is responsible for guiding the overall movement of the network towards the target direction. As the majority in the network, the follower has limited understanding of the overall task information of the network, mainly following the leader’s movement and gradually gathering. The above two roles initially stratify the network: the leader resides in the upper layer of the network, sending control information to the followers; the followers reside in the lower layer of the network, sending feedback and situational awareness information upwards, ultimately achieving circular flow of information within the network. From the analysis, it can be seen that the leader role in the Couzin-leader model is equivalent to the control node $N^{C}$ in VANETs, and the follower role is equivalent to the general action node $N^{MCH}$.

The four motion rules are repulsion, attraction, alignment, and homing. The repulsion rule functions to prevent collisions between adjacent nodes; the attraction rule functions to prevent the emergence of isolated nodes and to make the network as a whole tend towards cohesion; the alignment rule functions to make the speed and direction of movement of nodes tend towards consistency with those of their neighbors. Through these rules, the overall motion of the network can be characterized, while maintaining the relative independence of individual movements within the network, ultimately achieving mathematical modeling of the movement characteristics of self-organizing networks. Combining the actual characteristics of VANETs, the four motion rules are mathematically described.

Exclusion rule: Each moving node $N_{i}$ in VANETs maintains a safe distance $d_{Co}$ from its neighboring nodes to avoid collisions. When the distance $d_{ij}$ between any neighboring node $N_{j}$ and the node $N_{i}$ is less than or equal to the safe distance, the node $N_{i}$ will adjust its direction to move away from the neighboring node $N_{j}$. The set of neighboring nodes that node $N_{i}$ needs to avoid is denoted as $Nei^{d_{Co}}=\left\{N_{j}|d_{ij}\leq d_{Co},j\neq i\right\}$. The calculation formula for the exclusion direction vector ${\hat{O}}_{i}^{re}$ of node $N_{i}$ is:

(27) $$\begin{array}{l} O_{i}^{re}\left(t+\Delta t\right)=\left(\cos\theta_{i}\left(t+\Delta t\right),\sin\theta_{i}\left(t+\Delta t\right)\right)\\ =-\sum_{N_{j}\in Nei^{d_{Co}}}\frac{\left(x_{j}\left(t\right),y_{j}\left(t\right)\right)-\left(x_{i}\left(t\right),y_{i}\left(t\right)\right)}{\left\|\left(x_{j}\left(t\right),y_{j}\left(t\right)\right)-\left(x_{i}\left(t\right),y_{i}\left(t\right)\right)\right\|} \end{array}$$

(28) $${\hat{O}}_{i}^{re}\left(t+\Delta t\right)=\frac{O_{i}^{re}\left(t+\Delta t\right)}{\left\|O_{i}^{re}\left(t+\Delta t\right)\right\|}$$

In the formula, $\left\|O_{i}^{re}\left(t+\Delta t\right)\right\|$ represents the norm of the vector $O_{i}^{re}\left(t+\Delta t\right)$. After normalization, the collision avoidance direction vector of the node $N_{i}$ is now a unit vector ${\hat{O}}_{i}^{re}\left(t+\Delta t\right)$.

Attraction rule: If a node $N_{i}$ has no neighboring nodes within its safety distance, that is, $Nei^{d_{Co}}=\emptyset$. Nodes $N_{i}$ will tend to attract neighboring nodes $Nei^{d_{At}}=\left\{N_{j}|d_{Co}<d_{ij}\leq d_{At},j\neq i\right\}$ within the distance $\left(d_{Co},d_{At}\right]$, achieving network convergence. Under this attraction rule, the calculation formula for the attraction direction vector ${\hat{O}}_{i}^{at}$ of the node $N_{i}$ is:

(29) $$O_{i}^{at}\left(t+\Delta t\right)=\sum_{N_{j}\in Nei^{d_{At}}}\frac{\left(x_{j}\left(t\right),y_{j}\left(t\right)\right)-\left(x_{i}\left(t\right),y_{i}\left(t\right)\right)}{\left\|\left(x_{j}\left(t\right),y_{j}\left(t\right)\right)-\left(x_{i}\left(t\right),y_{i}\left(t\right)\right)\right\|}$$

(30) $${\hat{O}}_{i}^{at}\left(t+\Delta t\right)=\frac{O_{i}^{at}\left(t+\Delta t\right)}{\left\|O_{i}^{at}\left(t+\Delta t\right)\right\|}$$

Alignment rule: Nodes adjust their own direction angles to face the average direction of neighboring nodes. This rule provides a common forward direction for the entire VANETs, thereby achieving overall movement. The calculation formula for the alignment direction vector ${\hat{O}}_{i}^{al}$ of node $N_{i}$ is:

(31) $$O_{i}^{al}\left(t+\Delta t\right)=\frac{O_{i}\left(t\right)}{\left\|O_{i}\left(t\right)\right\|}+\sum_{N_{j}\in Nei^{d_{At}}}\frac{O_{j}\left(t\right)}{\left\|O_{j}\left(t\right)\right\|}$$

(32) $${\hat{O}}_{i}^{al}\left(t+\Delta t\right)=\frac{O_{i}^{al}\left(t+\Delta t\right)}{\left\|O_{i}^{al}\left(t+\Delta t\right)\right\|}$$

Homing rule: For the leader, namely the control node (denoted as $N_{i}$) in VANETs, it is required to guide the overall network towards a predetermined direction, which is called homing. In this case, the calculation formula for the homing direction vector of node $N_{i}$ is:

(33) $${O^{\prime }}_{i}\left(t+\Delta t\right)=\frac{{\hat{O}}_{i}^{re}\left(t+\Delta t\right)+{\hat{O}}_{i}^{at}\left(t+\Delta t\right)+{\hat{O}}_{i}^{al}\left(t+\Delta t\right)}{\left\|{\hat{O}}_{i}^{re}\left(t+\Delta t\right)+{\hat{O}}_{i}^{at}\left(t+\Delta t\right)+{\hat{O}}_{i}^{al}\left(t+\Delta t\right)\right\|}$$

(34) $${\hat{O}}_{i}^{ho}\left(t+\Delta t\right)=\frac{{O^{\prime }}_{i}\left(t+\Delta t\right)+\omega_{i}g}{\left\|{O^{\prime }}_{i}\left(t+\Delta t\right)+\omega_{i}g\right\|}$$

In the formula, g represents a unit vector, indicating the overall task information of VANETs, such as the directional information when the target area is located along the x axis $g=\left(1,0\right)$. $\omega_{i}$ is a weight term, $\omega_{i}=0$ indicating that the node has no understanding of the overall task information of VANETs and moves completely influenced by neighboring nodes; $\omega_{i}=1$ indicates that the node has partial understanding of the overall task information of VANETs and is influenced by both the overall task information and neighboring nodes; $\omega_{i}>1$ represents that the node prefers to complete the overall task of VANETs by influencing the movement of neighboring nodes. For the control node $N_{i}$ in VANETs, it is generally set $\omega_{i}>1$.

From the actual deployment of VANETs, it can be seen that at the initial moment of the task, the follower nodes are not completely unaware of the task information, but rather possess limited information. They continuously deepen their understanding and comprehension of the overall task during the task duration, ultimately facilitating the overall completion of the predetermined task by VANETs. Therefore, the paper assumes that the follower nodes are also applicable to the homing rule. At this time, the calculation formula for the direction vector ${\hat{O}}_{j}^{ho}$ sought by the follower node $N_{j}$ is:

(35) $${O^{\prime }}_{j}\left(t+\Delta t\right)=\frac{{\hat{O}}_{j}^{re}\left(t+\Delta t\right)+{\hat{O}}_{j}^{at}\left(t+\Delta t\right)+{\hat{O}}_{j}^{al}\left(t+\Delta t\right)}{\left\|{\hat{O}}_{j}^{re}\left(t+\Delta t\right)+{\hat{O}}_{j}^{at}\left(t+\Delta t\right)+{\hat{O}}_{j}^{al}\left(t+\Delta t\right)\right\|}$$

(36) $${\hat{O}}_{j}^{ho}\left(t+\Delta t\right)=\frac{{O^{\prime }}_{j}\left(t+\Delta t\right)+\omega_{j}g}{\left\|{O^{\prime }}_{j}\left(t+\Delta t\right)+\omega_{j}g\right\|}$$

(37) $$\omega_{j}=\frac{2t}{t_{mi}}+Ta_{ini}$$

In the formula, t represents the current time; $t_{mi}$ is the planned time for the task; $Ta_{ini}$ is a constant and $0<Ta_{ini}<1$, representing the limited task information grasped by the follower node at the initial time t=0; for the follower node $N_{j}$, its weight term $\omega_{j}$ takes values within an interval $\left[Ta_{ini},2+Ta_{ini}\right]$.

In summary, the updated direction vector $O_{i}\left(t+\Delta t\right)$ of VANETs nodes at time t+Δt can be obtained.

(38) $$O_{i}\left(t+\Delta t\right)=\frac{{\hat{O}}_{i}^{re}\left(t+\Delta t\right)+{\hat{O}}_{i}^{at}\left(t+\Delta t\right)+{\hat{O}}_{i}^{al}\left(t+\Delta t\right)+{\hat{O}}_{i}^{ho}\left(t+\Delta t\right)}{\left\|{\hat{O}}_{i}^{re}\left(t+\Delta t\right)+{\hat{O}}_{i}^{at}\left(t+\Delta t\right)+{\hat{O}}_{i}^{al}\left(t+\Delta t\right)+{\hat{O}}_{i}^{ho}\left(t+\Delta t\right)\right\|}$$

The consistency of the influence exerted by leaders on followers within VANETs is characterized by indicators $O_{Net}$:

(39) $$O_{Net}=\frac{1}{m}\sum_{i=1}^{m}O_{i}\cdot g$$

For $O_{Net}=-1$, it indicates that the direction of the follower is completely opposite to that of the leader; for $O_{Net}=0$, it indicates that the directions of the followers in VANETs are completely disordered; for $O_{Net}=1$, it indicates that the direction of the follower is completely aligned with that of the leader; for other situations $\left(-1<O_{Net}<1\right)$, it indicates that some followers in VANETs are moving in the opposite direction to the leader.

### 5.3. VANETs Node Location Update Algorithm Based on the Improved Couzin-Leader Model

The paper proposes a VANETs node location update algorithm based on the improved Couzin-leader model:

(1) Initialize the parameters of the improved Couzin-leader model, and set the safety distance $d_{Co}$, attraction distance $\left(d_{Co},d_{At}\right]$, leader weight term $\omega_{i}$, and overall task information vector g;

(2) Obtain all node information at the current moment t, including the number of nodes operating normally, node coordinates $CoN_{i}\left(t\right)$, node direction vectors $O_{i}\left(t\right)$, and node types (whether they are leaders or followers);

(3) Calculate the inter-node distance. Based on the node coordinate information $CoN_{i}\left(t\right)$, traverse all working node pairs $\left(i,j\right)$ within VANETs to generate an Euclidean distance matrix $M_{d}\left(t\right)$ between nodes, where i≠j;

(4) Identify the set of neighboring nodes, traverse all nodes within VANETs, and sequentially identify the set of neighboring nodes $Nei^{d_{Co}}=\left\{N_{j}|d_{ij}\leq d_{Co},j\neq i\right\}$ that $N_{i}$ needs to be avoided and the set of neighboring nodes $Nei^{d_{At}}=\left\{N_{j}|d_{Co}<d_{ij}\leq d_{At},j\neq i\right\}$ that $N_{i}$ are prone to attraction. If $Nei^{d_{Co}}\neq \emptyset$, proceed to step (5); otherwise, proceed to step (6);

(5) Apply the exclusion rule and substitute the exclusion rule formula to calculate the exclusion direction vector ${\hat{O}}_{i}^{re}$ of node $N_{i}$;

(6) Apply the attraction rule, and calculate the attraction direction vector ${\hat{O}}_{i}^{at}$ of node $N_{i}$;

(7) Apply the alignment rule, and calculate the alignment direction vector ${\hat{O}}_{i}^{al}$ of node $N_{i}$;

(8) Apply the homing rule, and calculate the direction vector ${\hat{O}}_{i}^{ho}$ of the node $N_{i}$;

(9) Update the node direction vector, and substitute it into the update equation to calculate the updated direction vector $O_{i}\left(t+\Delta t\right)$ of the node at the next moment t+Δt;

(10) Update the node speed and position, iterate through all nodes and substitute them into the position update equation, and calculate the node coordinates $CoN_{i}\left(t+\Delta t\right)$ at the next moment t+Δt;

(11) Iterate and repeatedly execute steps (2)–(10) of the algorithm until the termination condition is met (such as the number of normal working nodes reducing to 0, reaching the set task time $t_{mi}$, etc.).

### 5.4. Node Communication Link Modeling

According to the binary communication link model, a communication connection is established between nodes $N_{i}$ and $N_{j}$ if their Euclidean distance $d_{ij}\left(t\right)$ is less than the communication distance threshold $d_{Th}$. A time-varying binary communication connectivity adjacency matrix $A_{B}\left(t\right)$ is established:

(40) $$A_{B}\left(t\right)=\left[\begin{array}{cccc} a_{11}\left(t\right) & a_{12}\left(t\right) & \ldots & a_{1m}\left(t\right)\\ a_{21}\left(t\right) & a_{22}\left(t\right) & \ldots & a_{2m}\left(t\right)\\ \vdots & \vdots & \ddots & \vdots \\ a_{m1}\left(t\right) & a_{m2}\left(t\right) & \ldots & a_{mm}\left(t\right) \end{array}\right]$$

The elements $a_{ij}\left(t\right)=1\;\left(i\neq j\right)$ in the matrix represent that the nodes $N_{i}$ and $N_{j}$ are connected at time t. Based on the assumption of symmetry of communication links, there exists $a_{ij}\left(t\right)=a_{ji}\left(t\right)$. Where $a_{ij}=0\;\left(i\neq j\right)$ or $a_{ji}\left(t\right)=0$ indicates that the nodes $N_{i}$ and $N_{j}$ are not connected at time t, and node self-loops are not considered, that is:

(41) $$a_{ij}\left(t\right)=\left\{\begin{array}{ll} 1, & d_{ij}\left(t\right)\leq d_{Th},i\neq j\\ 0, & \mathrm{else} \end{array}\right.$$

Based on the aforementioned formula, the binary communication connectivity adjacency matrix $A_{B}\left(t\right)$ can be obtained by performing logical operations $A_{B}\left(t\right)=M_{d}\left(t\right)\leq d_{Th}$ on the Euclidean distance matrix $M_{d}\left(t\right)$ between nodes. This operation involves sequentially comparing the elements $d_{ij}\left(t\right)$ in the matrix $M_{d}\left(t\right)$ with real numbers $d_{Th}$: if $d_{ij}\left(t\right)\leq d_{Th}$ holds, the logical result is true, and a value of 1 is returned, with $a_{ij}\left(t\right)=1$; otherwise, the logical result is false, with $a_{ij}\left(t\right)=0$.

## 6. Analysis and Modeling of Intentional Attack

**Figure 4:**
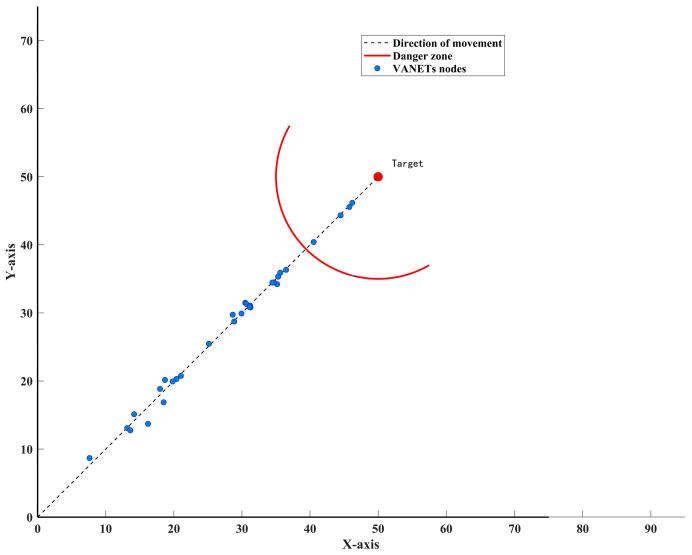
VANETs node movement and Danger zone.

From a risk management perspective, considering the potential dangers of VANETs in certain operational scenarios, nodes closer to the hazard source are more likely to be attacked. For instance, when VANETs advance toward a target fire area and conduct activities (e.g., personnel search and rescue, firefighting), the target area often presents hazards such as flames, explosions, high temperatures, and smoke. These hazards preferentially interfere with nodes within the hazard source’s coverage range and even cause node failures, as illustrated in Figure 4.

According to Figure 4, VANETs nodes that enter the danger zone first tend to experience intentional attacks earlier and with greater intensity. Therefore, it can be assumed that the intensity of intentional attack is negatively correlated with the distance $d_{iTa}$ from the node $N_{i}$ to the target point $N_{Ta}$. Let the coordinates of the target point $N_{Ta}$ be $CoN_{Ta}=\left(x_{Ta},y_{Ta}\right)$, then the Euclidean distance $d_{iTa}\left(t\right)$ between node $N_{i}$ and target point $N_{Ta}$ at time t can be calculated as:

(42) $$d_{iTa}\left(t\right)=\sqrt{{\left(x_{i}\left(t\right)-x_{Ta}\right)}^{2}+{\left(y_{i}\left(t\right)-y_{Ta}\right)}^{2}}$$

The purpose of this paper is to analyze the impact of intentional attack events based on relative distance and hazard source coverage on the connectivity reliability of VANETs. Drawing on existing studies [23,39,40,41,42], the intentional attack-induced failure of nodes is modeled as an exponential distribution, where the parameter $\lambda_{i}^{T}$ is inversely proportional to $d_{iTa}$:

(43) $$\left\{\begin{array}{l} \mathrm{Pr}\left(S_{N_{i}}\left(t\right)=4\right)=1-e^{-\lambda_{i}^{T}t}\\ \lambda_{i}^{T}=\frac{1}{d_{iTa}} \end{array}\right.$$

In the formula, $S_{N_{i}}\left(t\right)=4$ indicates that node $N_{i}$ is in a failed state due to intentional attack at time t; $\lambda_{i}^{T}$ is a parameter in the exponential distribution of intentional attack events; and t≥0 represents the working time of node’s. Consider $S_{N_{i}}\left(t\right)=4$ as an event $E_{N_{i}}^{4}\left(t\right)$, and the complementary event ${\bar{E}}_{N_{i}}^{4}\left(t\right)$ indicates that the node $N_{i}$ has not been subjected to an intentional attack at time t. Thus, we can conclude:

(44) $$\left\{\begin{array}{l} \mathrm{Pr}\left(E_{N_{i}}^{4}\left(t\right)\right)=\mathrm{Pr}\left(S_{N_{i}}\left(t\right)=4\right)=1-e^{-\lambda_{i}^{T}t}\\ \mathrm{Pr}\left({\bar{E}}_{N_{i}}^{4}\left(t\right)\right)=1-\mathrm{Pr}\left(E_{N_{i}}^{4}\left(t\right)\right)=e^{-\lambda_{i}^{T}t} \end{array}\right.$$

## 7. Analysis and Modeling of Node Isolation Failure

**Figure 5:**
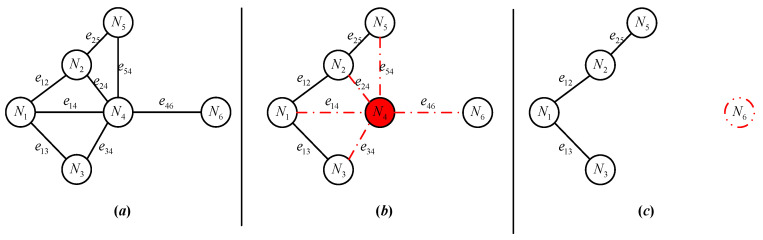
VANETs node isolation failure diagram; (**a**) the network topology at time a; (**b**) the network topology at time b; (**c**) the network topology at time c.

In VANETs, when multiple nodes fail simultaneously, it may result in a situation where the distance between a certain node $N_{i}$ and its nearest neighbor $N_{j}$ exceeds the communication distance threshold. That is $d_{ij}>d_{Th}$, the node $N_{i}$ cannot establish communication connectivity with the nearest neighbor, let alone connect with other nodes within the network, and remains in an isolated state. Although the node $N_{i}$ does not experience hardware/software failure, energy depletion, or intentional attack at this time, considering its loss of communication function and unavailability of message services within VANETs, it is still considered as failed and set to an isolated failure state S=5, as show in Figure 5.

In Figure 5a the network comprises 6 nodes and 8 edges, where communication links only exist between nodes $N_{6}$ and $N_{4}$. In Figure 5b When the node $N_{4}$ fails (such as running out of energy), it directly affects edges $e_{54}$, $e_{24}$, $e_{14}$, $e_{34}$ and $e_{46}$, which are marked with red dashed lines in the figure. In Figure 5c, after removing the failed node, the connectivity among the original four nodes $N_{1}$, $N_{2}$, $N_{3}$ and $N_{5}$ remains unchanged. However, since the Euclidean distance between the node $N_{6}$ and the four nodes exceeds the communication distance threshold, it cannot establish communication links with them. Even if the node $N_{6}$ does not experience hardware/software failures, energy depletion, intentional attacks, or other failure events, it is still defined as an isolated fault state and marked with a red dashed line, indicating that faults among VANETs nodes are not completely independent and exhibit a certain degree of correlation.

For the isolation failure event of VANETs nodes, it can be described through a time-varying binary communication connectivity adjacency matrix $A_{B}\left(t\right)$. Any node in a given state $S_{N_{i}}=1$, if it satisfies the following conditions at time t:

(45) $$\sum_{j\in V}a_{ij}=\sum_{j\in V}a_{ji}=0$$

That is, determine the isolation failure of the node $N_{i}$ at time t, record the node state $S_{N_{i}}\left(t\right)=5$, and record the event $E_{N_{i}}^{5}\left(t\right)$.

## 8. Connectivity Reliability Evaluation Algorithm

Based on the analysis presented in the paper, there are primarily four types of communication function failures in nodes during the actual deployment and operation of VANETs: hardware/software failures, energy consumption failures, intentional attacks, and isolation failures. Additionally, the high-speed mobility characteristics of nodes also impact communication connectivity. Considering that accurately solving the connectivity reliability of VANETs is an NP-hard problem, the paper proposes a simulation-based algorithm to approximately solve and evaluate the connectivity reliability of VANETs.

### 8.1. Connectivity Reliability Metrics Definition

1. Failure proportion of node failure mode

For the four VANETs node failure modes studied in the paper, the number of failed nodes under different failure modes is counted separately and compared with the total number of failed nodes. Based on this, the most prone failure mode among VANETs nodes is analyzed, providing a basis for subsequent optimization measures such as node improvement and protection.

Let $NF_{2}^{i}\left(t\right)$, $NF_{3}^{i}\left(t\right)$, $NF_{4}^{i}\left(t\right)$ and $NF_{5}^{i}\left(t\right)$ represent the number of failed nodes due to hardware/software failures, energy consumption failures, intentional attacks, and isolation failures in VANETs at time t in the ith simulation experiment, respectively; let $NF^{i}\left(t\right)$ represent the total number of failed nodes in VANETs at time t in the ith simulation experiment; $FP_{2}^{i}\left(t\right)$, $FP_{3}^{i}\left(t\right)$, $FP_{4}^{i}\left(t\right)$ and $FP_{5}^{i}\left(t\right)$ represent the proportions of failed nodes in VANETs at time t in the ith simulation experiment-due to hardware/software failures, energy consumption failures, intentional attacks, and isolation failures, respectively. Additionally,

(46) $$NF^{i}\left(t\right)=NF_{2}^{i}\left(t\right)+NF_{3}^{i}\left(t\right)+NF_{4}^{i}\left(t\right)+NF_{5}^{i}\left(t\right)$$

The formula for calculating the failure proportion $FP_{k}^{i}\left(t\right)$ of fault mode $FM_{k}\left(k=2,3,4,5\right)$ at time t in the ith simulation experiment is:

(47) $$FP_{k}^{i}\left(t\right)=\frac{NF_{k}^{i}\left(t\right)}{NF^{i}\left(t\right)}=\frac{NF_{k}^{i}\left(t\right)}{\sum_{j=2}^{5}NF_{j}^{i}\left(t\right)}$$

After running $N_{Simu}$ simulation experiments, the formula for calculating the average failure proportion $FP_{k}\left(t\right)$ of VANETs nodes’ failure modes $FM_{k}$ $\left(k=2,3,4,5\right)$ at time t is as follows:

(48) $$FP_{k}\left(t\right)=\frac{1}{N_{simu}}\sum_{i=1}^{N_{simu}}FP_{k}^{i}\left(t\right)=\frac{1}{N_{simu}}\sum_{i=1}^{N_{simu}}\frac{NF_{k}^{i}\left(t\right)}{NF^{i}\left(t\right)}$$

2. Residual energy of nodes

For the three types of nodes classified in the analysis of node energy consumption failure: control nodes $N^{C}=\left\{N_{i}^{C}|i=1,2,\ldots ,m_{1}\right\}$, convex hull nodes $N^{MCH}=\left\{N_{i}^{MCH}|i=1,2,\ldots ,m_{2}\right\}$, and general nodes $N^{MG}=\left\{N_{i}^{MG}|i=1,2,\ldots ,m_{3}\right\}$, the remaining energy of each type of nodes at different moments is statistically analyzed and compared. Based on this, the energy consumption patterns of different nodes are analyzed, providing a decision-making basis for VANETs node deployment and routing rule optimization.

Let $E_{C}^{i}\left(t\right)$, $E_{MCH}^{i}\left(t\right)$, $E_{MG}^{i}\left(t\right)$ and $E_{AN}^{i}\left(t\right)$ represent the remaining energy of the control nodes, convex hull nodes, general nodes, and all network nodes in VANETs at time t in the ith simulation experiment, respectively. Let $E_{C}\left(t\right)$, $E_{MCH}\left(t\right)$, $E_{MG}\left(t\right)$ and $E_{AN}\left(t\right)$ represent the remaining energy of the control nodes, convex hull nodes, general nodes, and all network nodes in VANETs at time t after running $N_{simu}$ simulation experiments, respectively. Then we have:

(49) $$\left\{\begin{array}{l} E_{C}^{i}\left(t\right)=\frac{1}{m_{1}}\sum_{i=1}^{m_{1}}E_{i}\left(t\right)\\ E_{MCH}^{i}\left(t\right)=\frac{1}{m_{2}}\sum_{i=1}^{m_{2}}E_{i}\left(t\right)\\ E_{MG}^{i}\left(t\right)=\frac{1}{m_{3}}\sum_{i=1}^{m_{3}}E_{i}\left(t\right)\\ E_{AN}^{i}\left(t\right)=\frac{1}{m_{AN}}\sum_{i=1}^{m_{AN}}E_{i}\left(t\right),m_{AN}=m_{1}+m_{2}+m_{3} \end{array}\right.$$

(50) $$\left\{\begin{array}{l} E_{C}\left(t\right)=\frac{1}{N_{simu}}\sum_{k=1}^{N_{simu}}E_{C}^{k}\left(t\right)=\frac{1}{N_{simu}\times m_{1}}\sum_{k=1}^{N_{simu}}\sum_{i=1}^{m_{1}}E_{i}\left(t\right)\\ E_{MCH}\left(t\right)=\frac{1}{N_{simu}}\sum_{k=1}^{N_{simu}}E_{MCH}^{k}\left(t\right)=\frac{1}{N_{simu}\times m_{2}}\sum_{k=1}^{N_{simu}}\sum_{i=1}^{m_{2}}E_{i}\left(t\right)\\ E_{MG}\left(t\right)=\frac{1}{N_{simu}}\sum_{k=1}^{N_{simu}}E_{MG}^{k}\left(t\right)=\frac{1}{N_{simu}\times m_{3}}\sum_{k=1}^{N_{simu}}\sum_{i=1}^{m_{3}}E_{i}\left(t\right)\\ E_{AN}\left(t\right)=\frac{1}{N_{simu}}\sum_{k=1}^{N_{simu}}E_{AN}^{k}\left(t\right) \end{array}\right.$$

3. Appointed end-to-end (s-t) connectivity reliability

In the ith simulation experiment, let the set of nodes within VANETs still possess normal communication functions at time t and can complete information receiving and transmitting tasks be denoted as $V_{1}\left(t\right)$, i.e., $V_{1}\left(t\right)=\left\{N_{i}|S_{N_{i}}=1,N_{i}\in V\right\}$. In the ith simulation experiment, let the number of nodes within $V_{1}\left(t\right)$ be denoted as $NV_{1}\left(t\right)$, then $NV_{1}\left(t\right)=\left|V_{1}\left(t\right)\right|$.

When VANETs need to ensure that the source node $N_{s}$ maintains communication connectivity with a specified sink (terminal) node $N_{t}$ during operation, as can be inferred from the aforementioned analysis, it is equivalent to the condition where the sink node $N_{t}$ experiences no faults, i.e., the sink node $N_{t}$ status value is $S_{N_{t}}=1$, and there exists a routing path between the source node $N_{s}$ and the sink node $N_{t}$. At this time, both the source node $N_{s}$ and the specified sink node $N_{t}$ must belong to the set $V_{1}$: the nodes are free from failures, their communication functions are normal, and there is no isolation failure, ensuring that a routing path always exists between the source nodes and the sink node. Therefore, to solve the connectivity reliability between two specified ends in VANETs through Monte Carlo simulation, it is necessary to check whether the source node $N_{s}$ and the specified sink node $N_{t}$ belong to the set $V_{1}\left(t\right)$ in each simulation time step. Introduce indicator functions $I_{N_{s}}^{V_{1}}\left(t\right)$ and $I_{N_{t}}^{V_{1}}\left(t\right)$:

(51) INsV1t=1,Ns∈V1t0,Ns∈V1t

(52) INtV1t=1,Nt∈V1t0,Nt∈V1t

In the formula, if the source node $N_{s}$ at time t belongs to the set $V_{1}\left(t\right)$, then $I_{N_{s}}^{V_{1}}\left(t\right)=1$; otherwise, $I_{N_{s}}^{V_{1}}\left(t\right)=0$. If the designated sink node $N_{t}$ at time t belongs to the set $V_{1}\left(t\right)$, then $I_{N_{t}}^{V_{1}}\left(t\right)=1$; otherwise, $I_{N_{t}}^{V_{1}}\left(t\right)=0$. After running $N_{simu}$ simulation experiments, the formula for calculating the average appointed end-to-end (s-t) connectivity reliability between two of VANETs at time t is:

(53) $$AppR_{st}\left(t\right)=\frac{1}{N_{simu}}\sum_{i=1}^{N_{simu}}I_{N_{s}}^{V_{1}}\left(t\right)I_{N_{t}}^{V_{1}}\left(t\right)$$

4. Arbitrarily end-to-end (s-t) connectivity reliability

When VANETs are operating and need to ensure that the source node $N_{s}$ always maintains communication connectivity with at least one sink node $N_{j}$ $\left(N_{j}\in V,j\neq s\right)$, it is equivalent to saying that there exists at least one sink node that is not experiencing any faults, i.e., the sink node $N_{j}$ status value is 1, and there is a route between it and the source node. At this time, the source node $N_{s}$ and at least one sink node $N_{j}$ must simultaneously belong to the set $V_{1}$. To solve the connectivity reliability of any two ends within VANETs through Monte Carlo simulation, it is necessary to check in each simulation time step t whether the source node $N_{s}$ and one arbitrarily sink node $N_{j}$ belong to the set $V_{1}\left(t\right)$. An indicator function $I_{2}^{V_{1}}\left(t\right)$ is introduced:

(54) $$I_{2}^{V_{1}}\left(t\right)=\left\{\begin{array}{l} 1,NV_{1}\left(t\right)\geq 2\\ 0,NV_{1}\left(t\right)<2 \end{array}\right.$$

In the formula, if the number of nodes in the set $V_{1}\left(t\right)$ normally communicating connected nodes at time t is greater than or equal to 2, the value is set to 1; otherwise, it is set to 0. After running $N_{simu}$ simulation experiments, the formula for calculating the average connectivity reliability between two arbitrarily ends in VANETs at time t is:

(55) $$ArbR_{st}\left(t\right)=\frac{1}{N_{simu}}\sum_{i=1}^{N_{simu}}I_{N_{s}}^{V_{1}}\left(t\right)I_{2}^{V_{1}}\left(t\right)$$

5. Appointed end-to-K ends (s-Kt) connectivity reliability

When VANETs need to ensure that the source node $N_{s}$ maintains communication connectivity with a specified number of K sink nodes $N_{t_{i}}$ $\left(i=1,2,\ldots ,K<m\right)$ during operation, as can be inferred from the aforementioned analysis, it is equivalent to assuming that none of the appointed K sink nodes $N_{t_{i}}$ experience any failures, and there exists a route between them and the source node $N_{s}$. At this point, both the source node $N_{s}$ and the appointed K sink nodes must simultaneously belong to the set $V_{1}$. An indicator function $I_{KN}^{V_{1}}\left(t\right)$ is introduced:

(56) $$I_{KN}^{V_{1}}\left(t\right)=\left\{\begin{array}{l} 1,N_{t_{i}}\in V_{1};i=1,2,\ldots ,K\\ 0,\mathrm{else} \end{array}\right.$$

In the formula, if the appointed K sink nodes $N_{t_{i}}$ at time t belongs to the set $V_{1}\left(t\right)$, then $I_{KN}^{V_{1}}\left(t\right)=1$, otherwise the value is 0. After running $N_{simu}$ simulation experiments, the formula for calculating the average appointed K terminal connectivity reliability of VANETs at time t is:

(57) $$AppR_{sKt}\left(t\right)=\frac{1}{N_{simu}}\sum_{i=1}^{N_{simu}}I_{N_{s}}^{V_{1}}\left(t\right)I_{KN}^{V_{1}}\left(t\right)$$

6. Arbitrarily end-to-K ends (s-Kt) connectivity reliability

When VANETs need to ensure that the source node $N_{s}$ always maintains communication with at least any K sink nodes $N_{j}$ $\left(N_{j}\in V;j=1,2,\ldots ,K<m;j\neq s\right)$ during operation, it is equivalent to the existence of at least K sink nodes without any failure, that is, the sink nodes status value are 1, and there is a route between every sink node and the source node. At this time, the source node and at least any K sink nodes must belong to the set $V_{1}$. To solve the arbitrarily end-to-K ends (s-Kt) connectivity reliability of VANETs through Monte Carlo simulation, it is necessary to check whether the source node and at least K sink nodes belong to the set $V_{1}\left(t\right)$ in each time step. Introducing the indicator function $I_{K+1}^{V_{1}}\left(t\right)$:

(58) $$I_{K+1}^{V_{1}}\left(t\right)=\left\{\begin{array}{l} 1,NV_{1}\left(t\right)\geq K+1\\ 0,NV_{1}\left(t\right)<K+1 \end{array}\right.$$

In the formula, if the number of nodes in the set $V_{1}\left(t\right)$ at time t is greater than or equal to K+1 (K sink nodes plus 1 source node), the value is set to 1; otherwise, it is set to 0. After running $N_{simu}$ simulation experiments, the formula for calculating the average arbitrarily K terminal connectivity reliability of VANETs at time t is:

(59) $$ArbR_{sKt}\left(t\right)=\frac{1}{N_{simu}}\sum_{i=1}^{N_{simu}}I_{N_{s}}^{V_{1}}\left(t\right)I_{K+1}^{V_{1}}\left(t\right)$$

7. ALL-terminal (s-At) connectivity reliability

Considering the scenario where all nodes within VANETs are required to be connected at any given time t, we introduce an indicator function $I_{m}^{V_{1}}\left(t\right)$:

(60) $$I_{m}^{V_{1}}\left(t\right)=\left\{\begin{array}{l} 1,NV_{1}\left(t\right)=m\\ 0,NV_{1}\left(t\right)<m \end{array}\right.$$

After running $N_{simu}$ simulation experiments, the formula for calculating the average all-terminal (end-to-all ends) connectivity reliability of VANETs at time t is:

(61) $$ArbR_{sAt}\left(t\right)=\frac{1}{N_{simu}}\sum_{i=1}^{N_{simu}}I_{m}^{V_{1}}\left(t\right)$$

### 8.2. Simulation Algorithm for Connectivity Reliability of VANETs Based on Node Motion Characteristics

In this subsection, the paper will elaborate on in detail the specific process of the simulation solution algorithm for the connectivity reliability of VANETs. To facilitate the reference to the key terms and notations appearing in the simulation algorithm, the paper summarizes the main notations used in the analysis of previous chapters in Table 4.

(1) Input VANETs tasks and related operation information: Node information processing and energy consumption parameters $E_{elec}$, $\varepsilon_{amp}$, $\gamma_{i}\left(t\right)$, $l_{C2}$, $l_{SC}$, $l_{CO}$, $T_{SC}$, $T_{CO}$; node movement parameters $\left[v_{\min},v_{\max}\right]$, $d_{Co}$, $d_{At}$, g, $\omega_{i}$; task information $Area_{mi}$, $t_{mi}$, $CoN_{Ta}$. Where $t_{mi}$ is the duration of VANETs task execution; $Area_{mi}$ is the area where VANETs execute tasks; $CoN_{Ta}=\left(x_{Ta},y_{Ta}\right)$ is the target point coordinate.

(2) Initialize VANETs graph theory model $G=\left(V,E\right)$ at time t=0: Based on task and deployment information, determine the number of nodes $m=\left|V\right|$ in VANETs, the corresponding index values $N_{i}$ $\left(i=1,2,\ldots ,m\right)$ for each node, and the index value for the control node $N^{C}$; assign initial coordinates $CoN_{i}\left(0\right)$, initial directions $O_{i}\left(0\right)$, and initial velocities $v_{i}\left(0\right)$ to all nodes; set up a set $V_{1}=V$ of nodes with normal communication functions, a set $V_{2}=\emptyset$ of nodes with hardware/software failures, a set $V_{3}=\emptyset$ of nodes with energy consumption failures, a set $V_{4}=\emptyset$ of nodes under attack, and a set $V_{5}=\emptyset$ of nodes with isolation failure.

(3) Determine whether a hardware/software failure event has occurred at nodes: For all nodes in the current $V_{1}$, calculate the probability $\mathrm{Pr}\left({\bar{E}}_{N_{i}}^{2}\left(t\right)\right)$ of each node not experiencing a hardware/software failure, and compare it with a random number 0≤r≤1. If $\mathrm{Pr}\left({\bar{E}}_{N_{i}}^{2}\left(t\right)\right)<r$, then a failure is determined, and updates $V_{1}=V_{1}\backslash \left\{N_{i}\right\}$, $V_{2}=V_{2}\cup \left\{N_{i}\right\}$ are made. After traversing all normal nodes in the current VANETs, if $V_{1}\neq \emptyset$, then proceed to step (4); otherwise, proceed to step (15) of the algorithm.

(4) Determine whether nodes have been intentional attacked and failed: For all nodes in the current $V_{1}$, calculate the probability $\mathrm{Pr}\left({\bar{E}}_{N_{i}}^{4}\left(t\right)\right)$ of each node not failing due to an intentional attack event, and compare it with a random number 0≤r≤1. If $\mathrm{Pr}\left({\bar{E}}_{N_{i}}^{4}\left(t\right)\right)<r$, then it is determined to have failed, update $V_{4}=V_{4}\cup \left\{N_{i}\right\}$, and update $V_{1}=V_{1}\backslash \left\{N_{i}\right\}$. After traversing all normal nodes in the current VANETs, if $V_{1}\neq \emptyset$, then proceed to step (5); otherwise, proceed to step (15) of the algorithm.

(5) Generate node distance matrix and adjacency matrix: For all nodes in the current $V_{1}$, generate the Euclidean distance matrix between nodes, and generate the binary communication connectivity adjacency matrix through logical operations $A_{B}\left(t\right)=M_{d}\left(t\right)\leq d_{Th}$.

(6) Update VANETs convex hull set: Keep the index values $N_{1}$ of control node $N^{C}$ unchanged, update the convex hull set $N^{MCH}\neq \emptyset$ and general action node set $N^{MG}\neq \emptyset$ based on the node coordinate information, and $V_{1}=\left\{N_{1}\right\}\cup N^{MCH}\cup N^{MG}$ holds.

(7) Determine the current VANETs information transmission situation: Compare the current time t with the transmission cycle. If $\mathrm{mod}\left(t,T_{SC}\times T_{CO}\right)=0$, then there are simultaneous ITS1, ITS2, and ITS3 in the VANETs; if $\mathrm{mod}\left(t,T_{SC}\right)=0$, then there are simultaneous ITS1 and ITS2 at the current time; if $\mathrm{mod}\left(t,T_{CO}\right)=0$, then there is only ITS3 at the current time;

(8) Determine routing paths within VANETs: Apply routing path identification algorithms for different information transmission situations (ITS1, ITS2, ITS3) to determine all existing routing paths within the current VANETs.

(9) Calculating the information processing capacity of VANETs nodes: The formula for information processing capacity of nodes is applied to calculate the amount of information sent and received by each node.

(10) Determine whether nodes have experienced an energy consumption failure event: Calculate the remaining energy of all nodes in VANETs at the current moment t, and compare it with the energy threshold $E_{Th}$. If $E_{i}\left(t\right)<E_{Th}$, then it is determined as failure, update $V_{3}=V_{3}\cup \left\{N_{i}\right\}$, and update $V_{1}=V_{1}\backslash \left\{N_{i}\right\}$. After traversing all normal nodes in the current VANETs, if $V_{1}\neq \emptyset$, then proceed to step (11); otherwise, proceed to step (15) of the algorithm.

(11) Update distance matrix and adjacency matrix: For all nodes in the current $V_{1}$, generate the Euclidean distance matrix between nodes, and generate the binary communication connectivity adjacency matrix through logical operations $A_{B}\left(t\right)=M_{d}\left(t\right)\leq d_{Th}$.

(12) Determine whether an isolation failure event has occurred at nodes: Check all nodes in the system, if there is a node that satisfies $\sum_{j\in V_{1}}a_{ij}=\sum_{j\in V_{1}}a_{ji}=0$, it is determined that an isolation failure has occurred. Update $V_{5}=V_{5}\cup \left\{N_{i}\right\}$, update $V_{1}=V_{1}\backslash \left\{N_{i}\right\}$, and set $N^{MCH}=N^{MG}=\emptyset$. If $V_{1}\neq \emptyset$, proceed to step (13); otherwise, proceed to step (15).

(13) Update node location information: For all nodes in the current $V_{1}$, run the VANETs node location update algorithm based on the improved Couzin-leader model, calculate and update the direction vectors $O_{i}\left(t+\Delta t\right)$, coordinates $CoN_{i}\left(t+\Delta t\right)$, and other information of each node at the next moment t+Δt;

(14) Update the simulation clock: t=t+Δt, if $t\leq t_{mi}$, proceed to step (3) of the algorithm and continue the iterative operation; otherwise, proceed to step (15) of the algorithm.

(15) End the simulation algorithm and output data: the number of failed nodes $NF_{2}^{i}\left(t\right)$, $NF_{3}^{i}\left(t\right)$, $NF_{4}^{i}\left(t\right)$ and $NF_{5}^{i}\left(t\right)$; the remaining energy of nodes $E_{C}^{i}\left(t\right)$, $E_{MCH}^{i}\left(t\right)$ and $E_{MG}^{i}\left(t\right)$; the indicator function $I_{N_{s}}^{V_{1}}$, $I_{N_{t}}^{V_{1}}$, $I_{2}^{V_{1}}\left(t\right)$, $I_{KN}^{V_{1}}\left(t\right)$, $I_{K+1}^{V_{1}}\left(t\right)$, $I_{m}^{V_{1}}\left(t\right)$, etc.

## 9. Case Study

### 9.1. Simulation Case Parameter Setting

The paper illustrates the network connectivity reliability evaluation method proposed in the study through a specific VANETs application case. In this case, VANETs consists of 150 mobile nodes, including one control node that plays the role of a leader, and the remaining nodes play the role of followers. The network needs to proceed towards a predetermined target point, with a mission duration (single simulation duration) of 1 h. During the deployment phase, node coordinates, velocities, and directions are initialized. During the simulation experiment, motion information, node reliability information, link information, and network topology information are continuously updated. After repeating the simulation experiment 50 times, the network connectivity reliability parameters are estimated through average values. The relevant parameters of VANETs are shown in Table 5. The simulation experiments were completed using MATLAB R2023.

### 9.2. Simulation Case Result Analysis

**Figure 6:**
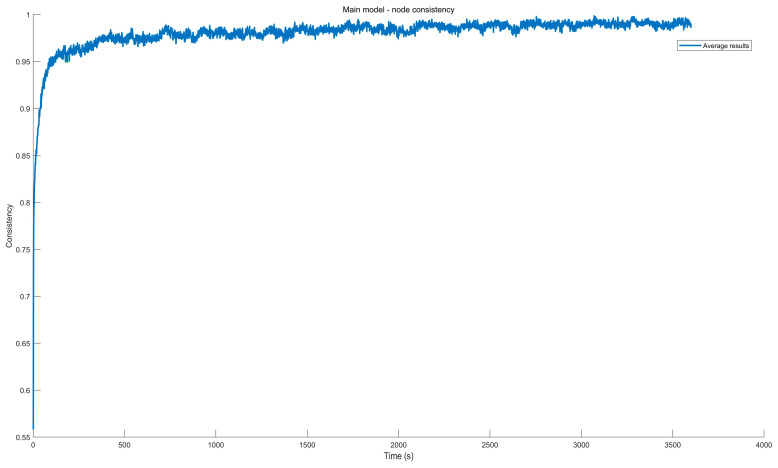
Node consistency analysis of the main model.

The simulation model utilizing the proposed routing path recognition algorithm is named as the main model, while the simulation model without utilizing the routing path recognition algorithm serves as the control model. Both models underwent 50 simulation experiments, respectively. Figure 6 illustrates the node consistency analysis of the main model. It can be observed that due to the random deployment of nodes within VANETs at the initial moment, $O_{Net}\left(0\right)=0.5582$, as the simulation time progresses, follower nodes are influenced by the leader node, continuously enhancing the overall movement consistency of the cluster and moving towards the target area. At time t=436, $O_{Net}\left(t\right)$ begins to converge to the range $\left[0.9761,\;0.9975\right]$, reflecting a high degree of organization and discipline during the task process, proving the effectiveness of the improved Couzin-leader model proposed in this paper.

**Figure 7:**
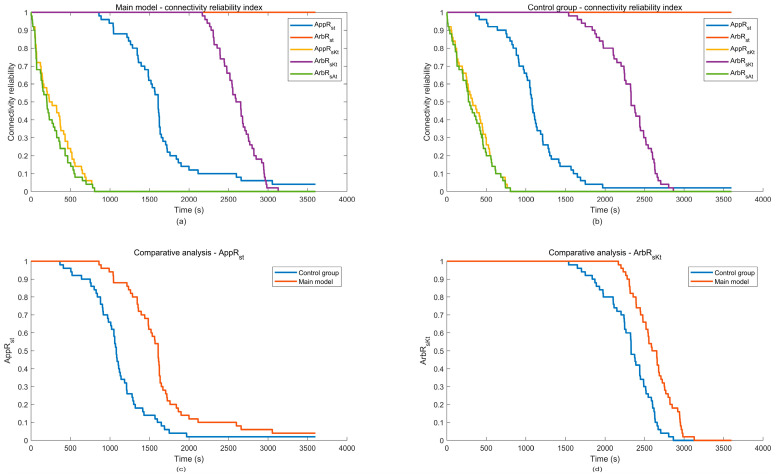
Evaluation values of connectivity reliability metrics in simulation experiments; (**a**) connectivity reliability metrics of the main model; (**b**) connectivity reliability metrics of the control model; (**c**) $AppR_{st}\left(t\right)$ of the main model and control model; (**d**) $ArbR_{sKt}\left(t\right)$ of the main model and control model; K = 120.

Figure 7 shows the comparison of evaluation values for connectivity reliability metrics.

From Figure 7a,b, it can be observed that among the connectivity reliability evaluation metrics of VANETs, there exists a pattern where $ArbR_{sAt}\left(t\right)$ < $AppR_{sKt}\left(t\right)$ < $AppR_{st}\left(t\right)$ < $ArbR_{sKt}\left(t\right)$ < $ArbR_{st}\left(t\right)$ at the same time, with K=120. Furthermore, both $ArbR_{sAt}\left(t\right)$ and $AppR_{sKt}\left(t\right)$ decrease rapidly after the start of the simulation experiment, which is observed in both the main model and the control model. Specifically, $ArbR_{sAt}\left(805\right)=AppR_{sKt}\left(805\right)=0$ in both models. In contrast, $ArbR_{st}\left(t\right)$ remains 1 even at the end of the simulation experiment (i.e., $ArbR_{st}\left(3600\right)=1$), indicating that the above 3 connectivity reliability metrics have low value for practical management decisions.

This paper further focuses on two metrics: $AppR_{st}\left(t\right)$ and $ArbR_{sKt}\left(t\right)$, as shown in Figure 7c,d. It can be seen that at any given time, the evaluation value of the main model is greater than that of the control model. Taking $AppR_{st}\left(t\right)$ as an example, when it decreases to 0.8, the corresponding time for the control model is t=840, while the corresponding time for the main model is t=1285, indicating an increase of 52.98% in VANETs working time compared to the former. When it decreases to 0.6, the corresponding time for the control model is t=1050, while the corresponding time for the main model is t=1520, indicating an increase of 44.76% in VANETs working time compared to the former. Taking $ArbR_{sKt}\left(t\right)$ as an example, when it decreases to 0.8, the corresponding time for the control model is t=1978, while that for the main model is t=2356. This indicates an increase of 19.11% in VANETs working time compared to the former. When it decreases to 0.6, the corresponding time for the control model is t=2265, while the corresponding time for the main model is t=2543, indicating an increase of 12.27% in VANETs working time compared to the former.

**Figure 8:**
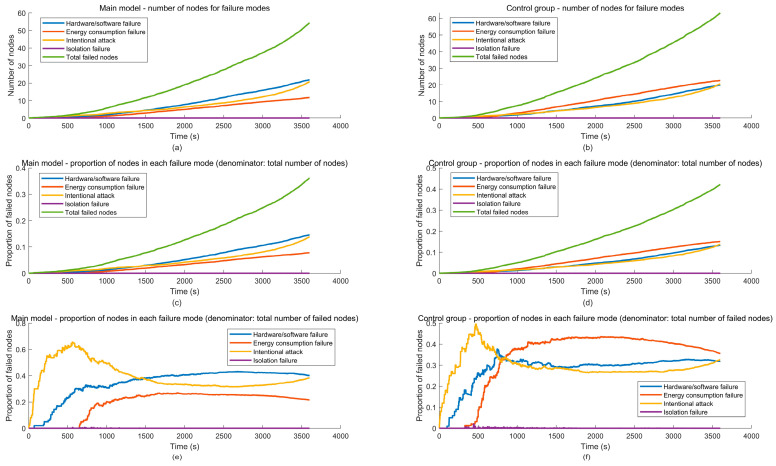
Analysis of various fault modes; (**a**) the number of various types of failed nodes for the main model; (**b**) the number of various types of failed nodes for the control model; (**c**) the proportion of various types of failed nodes for the main model (denominator: the total number of nodes); (**d**) the proportion of various types of failed nodes for the control model (denominator: the total number of nodes); (**e**) the proportion of various types of failed nodes for the main model (denominator: the total number of failed nodes); (**f**) the proportion of various types of failed nodes for the control model (denominator: the total number of failed nodes).

When distinguish between the main model and the control model, various failure modes during VANETs operation are analyzed, as shown in Figure 8 specifically.

According to Figure 8a,b, the total number of failed nodes increases at an accelerating rate as the simulation time progresses. For both the main model and the control model, no isolation failure mode occurred during the simulation experiment. However, the main failure modes differed in the simulation experiments: in the control model, there was $NF_{3}\left(3600\right)=22.6$ > $NF_{4}\left(3600\right)=20.68$ > $NF_{2}\left(3600\right)=20.02$, and the main failure mode was energy consumption failure, with a total number of failed nodes $NF\left(3600\right)=63.3$; while in the main model, there was $NF_{2}\left(3600\right)=21.84$ > $NF_{4}\left(3600\right)=20.8$ > $NF_{3}\left(3600\right)=11.68$, and the main failure mode was hardware/software failure, with a total number of failed nodes $NF\left(3600\right)=54.32$. At the end of the simulation experiment, the frequency of four failure events is listed in Table 6. Figure 8c,d is equivalent to scaling Figure 8a,b on the y-axis (in the example m), thus the variation pattern is consistent. It should be noted that although the main model has a 14.19% reduction in the total number of failed nodes compared to the control model, in fact, the main model only has one failure mode of energy consumption failure with a smaller number of failed nodes than the control model (a decrease of 48%), while the number of failed nodes due to hardware/software failure increased by 9.1%, and the number of failed nodes due to intentional attacks increased by 0.58%. The above indicates that in VANETs operation, when the probability of energy consumption failure occurring in nodes decreases, there is even a higher probability (compared to the control model) of other failure modes occurring, rather than maintaining a working or operational state throughout.

Further analysis from Figure 8e,f reveals the occurrence pattern of failure modes within VANETs. In the control experiment, there is a clear turning point t=859. Before this point, the main failure mode among all failed nodes is intentional attack, followed by hardware/software failure, and finally energy consumption failure. After the turning point t=859, energy consumption failure becomes the main failure mode but its proportion tends to stabilize with a certain downward trend, while hardware/software failure remains the second largest failure mode with no fluctuation in proportion, and intentional attack shows a certain upward trend. In the main model, the turning point occurs before t=1469 which the failure occurrence pattern is similar to that in the control model. However, after the turning point, hardware/software failure becomes the main failure mode with a stable proportion, intentional attack shows an upward trend as the second largest failure mode, and energy consumption failure shows a certain downward trend. From the beginning of the simulation experiment to the turning point, node hardware/software failures follow an exponential distribution, and the cumulative amount of information sent and received is small, resulting in a low occurrence rate of these two failure modes. However, from the turning point to the end of the simulation experiment, as nodes continue to approach the target area, the probability of being attacked increases exponentially, leading to a significant increase in the intentional attack failure mode; for energy consumption failure, on the one hand, as the simulation time steps forward, the cumulative amount of information sent and received by nodes continues to increase, resulting in a decrease in the remaining energy of nodes and an increase in the probability of energy consumption failure. On the other hand, the total number of failed nodes continues to increase, leading to fewer and fewer nodes capable of sending and receiving information within the VANETs, and the instantaneous amount of information that nodes need to process continues to decrease, reducing the probability of energy consumption failure. The above analysis has a comprehensive impact on energy consumption failure, which is reflected in Figure 8e,f.

**Figure 9:**
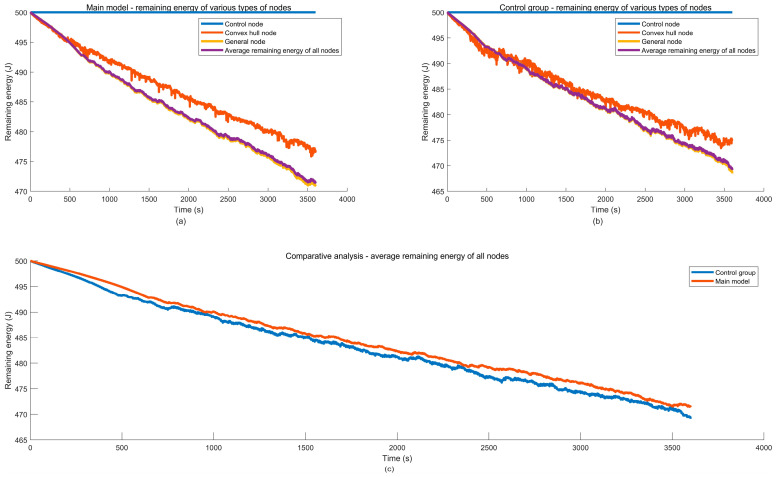
The remaining energy of VANETs nodes; (**a**) the remaining energy curves of four types of nodes in the main model; (**b**) the remaining energy curves of four types of nodes in the control model; (**c**) a comparative analysis of the average remaining energy of all network nodes in the main model and the control model.

Distinguishing between the main model and the control model, we analyze the remaining energy of nodes during the operation of VANETs, as shown in Figure 9. Since the control nodes are assumed to be fault-free, their residual energy remains constant. From Figure 9a,b, it can be observed that during the operation of VANETs, $E_{MG}\left(t\right)<E_{AN}\left(t\right)<E_{MCH}\left(t\right)$ holds. General nodes consume energy the fastest, followed by the average energy consumption of all network nodes, and convex hull nodes consume energy the slowest. From Figure 9c, it can be seen that at the end of the simulation experiment, the average remaining energy $E_{AN}\left(3600\right)=471.3736$ of all network nodes in the main model increased by 0.41% compared with that in the control model, $E_{AN}\left(3600\right)=469.1390$. Although this numerical difference is not significant, it still contributes to the reduction in energy consumption failure.

### 9.3. Sensitivity Analysis

Given that scholars have already conducted sensitivity analyses on network characteristics such as task areas, radio coverage, and network scale (number of nodes) [22,35], this paper further focuses on the cluster movement characteristics exhibited by VANETs in practical deployments, studying the impact of the nodes’ attraction distance on VANETs connectivity reliability. The repulsion distance $d_{Co}$ and communication distance thresholds $d_{Th}$ within a VANET are kept constant, and only the attraction distance $d_{At}$ is varied, with its value ranging within an interval $\left(d_{Co},d_{Th}\right]$. A new independent variable, Attractive-communication distance threshold ratio (ATR), is defined:

(62) $$ATR=\frac{d_{At}}{d_{Th}}$$

Each change in the value of the attraction distance $d_{At}$ will generate a new ATR, with $ATR\in \left(\frac{d_{Co}}{d_{Th}},1\right]$. For the simulation case proposed in this paper, the influence of different $d_{At}$ values $\left[30,\;40,\;60,\;80,\;100\right]$ on the connectivity reliability of VANETs is analyzed, that is, ATR values $\left[0.3,\;0.4,\;0.6,\;0.8,\;1\right]$, in which 50 simulation experiments are repeated for each ATR value, and the average result is used for subsequent analysis.

**Figure 10:**
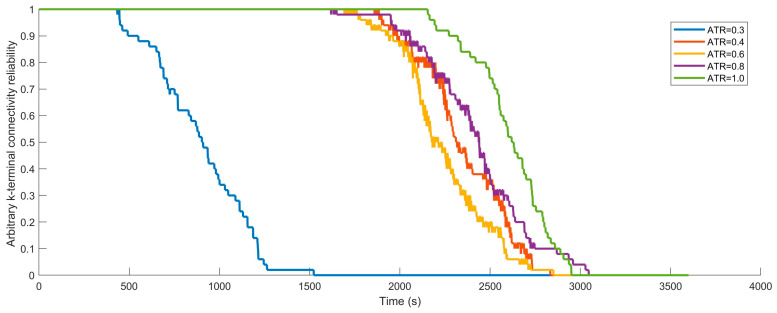
Impact of ATR on the connectivity reliability of VANETs.

Figure 10 illustrates the impact of different ATR on the $ArbR_{sKt}\left(t\right)$ of VANETs. It can be observed that at the same time, as the ATR increases, the $ArbR_{sKt}\left(t\right)$ of VANETs also shows an increasing trend, indicating ATR has a positive correlation with the connectivity reliability of VANETs.

**Figure 11:**
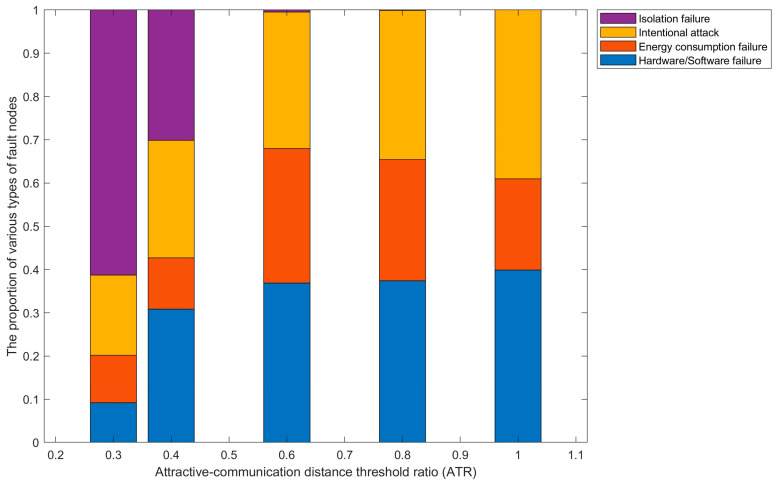
The proportion of various types of fault nodes varies with ATR at the end of the simulation experiment.

Further analyze the occurrence patterns of failure events for various types of fault nodes, as shown in Figure 11.

From Figure 11, it is evident that when ATR<0.6, the VANETs node has isolation failure during operation. However, in the previous main model simulation experiment, the isolation failure mode was not observed for ATR=1 was set. Furthermore, it can be observed that the proportion of isolation failures is extremely sensitive to changes in the ATR: when the ratio changes from 0.3 to 0.4, the proportion of isolation failures decreases sharply from 61.29% to 30.15% (the denominator represents the total number of failed nodes); when the ratio changes from 0.4 to 0.6, the proportion of isolation failures decreases sharply from 30.15% to 0.50%. In addition, it can be found that when there are isolation failure events in VANETs, the proportion of node energy consumption failures is relatively low, as a large number of isolated nodes do not perform information reception or transmission operations, resulting in lower energy consumption. This result further confirms the effectiveness and correctness of the simulation experiments in the paper.

## 10. Conclusions

The paper focuses on the connectivity reliability evaluation of VANETs, conducting research from aspects such as node failure modeling, mobility model construction, and connectivity reliability solving algorithm design. The specific content includes: analyzing four types of failure modes existing in VANETs nodes based on the assumption of imperfect nodes; analyzing the motion characteristics of VANETs clusters based on the node control relationships that exist in actual operation; proposing a routing path identification algorithm that considers energy consumption; and proposing a connectivity reliability evaluation algorithm for VANETs through the mapping relationship between mathematical models and simulation models.

The connectivity reliability solving algorithm proposed in the paper is verified through simulation examples, and it is demonstrated that the routing path identification algorithm considering energy consumption reduces the amount of information processed by nodes, thereby reducing node energy consumption and ultimately leading to a decrease in both the number and probability of energy consumption failures occurring in nodes. However, there is a correlation between different failure modes similar to competitive failure: a decrease in the probability of one failure mode may lead to more nodes failing due to other reasons. As the simulation time progresses, the occurrence patterns of different failure modes also vary, and the same failure mode (taking energy consumption failure as an example) may have both promoting and inhibiting factors. According to the sensitivity analysis, with the change of ATR, the most obvious change in VANETs is the occurrence of node isolation failure events: in this paper, when ATR=0.6, the proportion of isolation failure is only 0.50%, which can be predicted that when ATR>0.6, VANETs will not have isolation failure during operation. The limitations of this study lie in modeling the link reliability in VANETs using a binary communication model, which simplifies real-world scenarios and fails to consider the relationship between link capacity and node information processing load. In the future, the FS-TRG model will be introduced to account for complex scenarios such as signal fading and interference in VANETs node communication. Additionally, the impact of link capacity (which follows a certain probability distribution) on node information transmission will be considered to comprehensively analyze the problem of connectivity reliability evaluation for VANETs under the assumption of unreliable nodes and unreliable links. Finally, optimization suggestions will be proposed targeting the main sensitive factors.

## Figures and Tables

**Table 1:** Comparison of related work and this paper.

Literature	Research Object	Evaluation Indicators	Node Failure Modes	Link Connection Model	Mobility Model
[20]	VANETs	Reliability, survivability	Hardware failures	Probabilistic	/ (Simplified)
[28]	VANETs	Communication reliability	/ (Not considered)	Probabilistic	Gauss-Markov mobility model
[29]	VANETs	Network performance	/ (Not considered)	Deterministic	Microscopic mobility model
[30]	VANETs	Reliability	/ (Not considered)	Deterministic	Car-Following Model
[31]	VANETs	Network performance	/ (Not considered)	Deterministic	Realistic model of vehicular traffic
[32]	VANETs	Quality of Service (QoS) requirements	/ (Not considered)	Probabilistic	Macroscopic mobility model
[33]	VANETs	Reliability, resilience	Cascading failures	Deterministic	/ (Fixed nodes)
[34]	WSNs	Invulnerability	Intrinsic failure, external attack	Deterministic	/ (Fixed nodes)
[27]	MANET	Transmission reliability	/ (Not considered)	Probabilistic	Random direction mobility model
[35]	MANET	Connectivity reliability	Undefined but considered	Probabilistic	Random waypoint mobility model
[21]	WSNs	Transmission reliability	Random failure, energy consumption failure	Probabilistic	/ (Fixed nodes)
Ours	VANETs	Connectivity reliability	Hardware/software failure, energy consumption failure, intentional attack, isolation failure	Deterministic	Improved Couzin-leader model

**Table 2:** Correspondence between VANET node types and transmitted and received information.

Node Type	Generate and Send Information Type	Cycle	Received Information Type	Relay Information Type
$N^{C}$	$l_{C2}$	$T_{SC}$	$l_{SC}$ $,\;l_{CO}$	/
$N^{MCH}$	$l_{SC}$ $,\;l_{CO}$	$T_{SC}$	$l_{C2}$	/
$N^{MG}$ (Relay)	$l_{CO}$	$T_{CO}$	$l_{C2}$	$l_{SC}$ $,\;l_{C2}$ $,\;l_{CO}$
$N^{MG}$ (Non-relay)	$l_{CO}$	$T_{CO}$	$l_{C2}$	/

**Table 3:** Three information transmission situations (ITS) within VANETs.

ITS	Source Node	Sink Nodes	Delivery Cycle	Send Information Type
ITS1	$N^{C}$	$N^{MCH}$, $N^{MG}$	$T_{SC}$	$l_{C2}$
ITS2	$N^{MCH}$	$N^{C}$	$T_{SC}$	$l_{SC}$
ITS3	$N^{MCH}$ $,\;N^{MG}$	$N^{C}$	$T_{CO}$	$l_{CO}$

**Table 4:** Notations.

Notation	Description
$E_{elec}$	the energy consumption of the circuit for transmitting 1 bit of information
$\varepsilon_{amp}$	the power consumption coefficient of the power amplification circuit
$\gamma_{i}\left(t\right)$	the interference factor of the environment at time t
$l_{C2}$	the control information
$l_{SC}$	the situational awareness information
$l_{CO}$	the coordinate information
$l_{T}^{N_{i}}\left(t\right)$	$\mathrm{the}\;\mathrm{amount}\;\mathrm{of}\;\mathrm{information}\;\mathrm{sent}\;\mathrm{by}\;\mathrm{the}\;\mathrm{node}\;N_{i}$ at time t
$l_{R}^{N_{i}}\left(t\right)$	$\mathrm{the}\;\mathrm{amount}\;\mathrm{of}\;\mathrm{information}\;\mathrm{received}\;\mathrm{by}\;\mathrm{the}\;N_{i}$ at time t
$E_{T}^{N_{i}}\left(t\right)$	$\mathrm{the}\;\mathrm{energy}\;\mathrm{consumed}\;\mathrm{by}\;\mathrm{node}\;N_{i}$ $\mathrm{to}\;\mathrm{send}\;\mathrm{information}\;l_{T}^{N_{i}}\left(t\right)$ to a distance d at time t
$E_{R}^{N_{i}}\left(t\right)$	$\mathrm{the}\;\mathrm{energy}\;\mathrm{consumed}\;\mathrm{by}\;\mathrm{node}\;N_{i}$ $\mathrm{to}\;\mathrm{receive}\;\mathrm{information}\;l_{R}^{N_{i}}\left(t\right)$ at time t
$T_{SC}$	the interval period for real-time control information to be sent
$T_{CO}$	the interval period for coordinate information to be sent
$v_{\min}$	the minimum velocity of nodes
$v_{\max}$	the maximum velocity of nodes
$d_{Co}$	the safety distance between nodes to avoid collision
$d_{At}$	the mutual attraction distance between nodes
g	a unit vector, indicating the overall task information of VANETs
$\omega_{i}$	a weight term to measure the degree of knowledge of task information
$Area_{mi}$	the area where VANETs execute tasks
$t_{mi}$	the duration of VANETs task execution
$CoN_{Ta}$	$CoN_{Ta}=\left(x_{Ta},y_{Ta}\right)$ is the target point coordinate
m	$m=\left|V\right|$ is the number of nodes in VANETs
$N_{i}$	$\mathrm{the}\;\mathrm{corresponding}\;\mathrm{index}\;\mathrm{value}\;\mathrm{for}\;\mathrm{the}\;i\mathrm{th}\;\left(i=1,2,\ldots ,m\right)$ node
$N^{C}$	the control node
$N^{MCH}$	the convex hull set
$N^{MG}$	the action node set
$CoN_{i}\left(t\right)$	$\mathrm{the}\;\mathrm{coordinate}\;\mathrm{of}\;\mathrm{node}\;N_{i}$ at time $t\;\left(t=0,1,\ldots ,t_{mi}\right)$
$O_{i}\left(t\right)$	$\mathrm{the}\;\mathrm{direction}\;\mathrm{of}\;\mathrm{node}\;N_{i}$ at time t
$v_{i}\left(t\right)$	$\mathrm{the}\;\mathrm{velocity}\;\mathrm{of}\;\mathrm{node}\;N_{i}$ at time t
$V_{1}$	the set of nodes with normal communication functions
$V_{2}$	the set of nodes with hardware/software failure
$V_{3}$	the set of nodes with energy consumption failure
$V_{4}$	the set of nodes under attack
$V_{5}$	the set of nodes with isolation failure
0≤r≤1	a random number
$\mathrm{Pr}\left({\bar{E}}_{N_{i}}^{j}\left(t\right)\right)$	$\mathrm{the}\;\mathrm{probability}\;\mathrm{of}\;\mathrm{node}\;N_{i}$ not experiencing the jth failure at time t, while j=2 indicates hardware/software failure, j=3 indicates energy consumption failure, j=4 indicates intentional attack, j=5 indicates isolation failure
$M_{d}\left(t\right)$	the Euclidean distance matrix between nodes in VANETs
$A_{B}\left(t\right)$	the binary communication connectivity adjacency matrix of VANETs
$a_{ij}$	$\mathrm{the}\;\mathrm{element}\;\mathrm{of}\;A_{B}\left(t\right)$
$E_{i}\left(t\right)$	$\mathrm{the}\;\mathrm{remaining}\;\mathrm{energy}\;\mathrm{of}\;\mathrm{node}\;N_{i}$
$E_{Th}$	the energy threshold
Δt	the simulation time step
$NF_{j}^{i}\left(t\right)$	the number of nodes failed in the jth failure mode in the ith simulation experiment
$I_{N_{s}}^{V_{1}}$	$\mathrm{the}\;\mathrm{indicator}\;\mathrm{function}\;\mathrm{for}\;\mathrm{judging}\;\mathrm{whether}\;\mathrm{the}\;\mathrm{source}\;\mathrm{node}\;N_{s}$ works normally at time t
$I_{N_{t}}^{V_{1}}$	$\mathrm{the}\;\mathrm{indicator}\;\mathrm{function}\;\mathrm{for}\;\mathrm{judging}\;\mathrm{whether}\;\mathrm{the}\;\mathrm{designated}\;\sin k\;\mathrm{node}\;N_{t}$ works normally at time t
$I_{2}^{V_{1}}\left(t\right)$	$\mathrm{the}\;\mathrm{indicator}\;\mathrm{function}\;\mathrm{for}\;\mathrm{judging}\;\mathrm{whether}\;\mathrm{the}\;\mathrm{number}\;\mathrm{of}\;\mathrm{nodes}\;\mathrm{in}\;\mathrm{the}\;\mathrm{set}\;V_{1}\left(t\right)$ normally communicating connected nodes at time t is greater than or equal to 2
$I_{KN}^{V_{1}}\left(t\right)$	the indicator function for judging the appointed K $\mathrm{sink}\;\mathrm{nodes}\;N_{t_{i}}\;\left(i=1,2,\ldots ,K<m\right)$ at time t $\mathrm{belongs}\;\mathrm{to}\;\mathrm{the}\;\mathrm{set}\;V_{1}\left(t\right)$
$I_{K+1}^{V_{1}}\left(t\right)$	$\mathrm{the}\;\mathrm{indicator}\;\mathrm{function}\;\mathrm{for}\;\mathrm{judging}\;\mathrm{whether}\;\mathrm{the}\;\mathrm{number}\;\mathrm{of}\;\mathrm{nodes}\;\mathrm{in}\;\mathrm{the}\;\mathrm{set}\;V_{1}\left(t\right)$ at time t is greater than or equal to K+1
$I_{m}^{V_{1}}\left(t\right)$	$\mathrm{the}\;\mathrm{indicator}\;\mathrm{function}\;\mathrm{for}\;\mathrm{judging}\;\mathrm{whether}\;\mathrm{the}\;\mathrm{number}\;\mathrm{of}\;\mathrm{nodes}\;\mathrm{in}\;\mathrm{the}\;\mathrm{set}\;V_{1}\left(t\right)$ at time t is equal to m

**Table 5:** VANET parameter settings.

Parameter	Value	Unit	Parameter	Value	Unit
$d_{At}$	100	m	$T_{CO}$	10	s
$d_{Co}$	20	m	E0	500	J
$d_{Th}$	100	m	MTTF	10,000	h
$v_{\min}$	20	km/h	Δt	1	s
$v_{\max}$	40	km/h	$CoN_{Ta}$	(19,000, 19,000)	m
$E_{elec}$	100 × 10^−9^	J/bit	g	[1, 1]	/
$\varepsilon_{amp}$	100 × 10^−12^	J/bit/m^2^	$\omega_{1}$	3	/
γ	2	/	$Ta_{ini}$	1	/
$l_{C2}$	10	KB	Appt	5	/
$l_{SC}$	10	KB	k	120	/
$l_{CO}$	4	KB	Appk	2:k+1	/
$T_{SC}$	5	s			

**Table 6:** Ranking of four types of failure modes.

Ranks	Experimental Model	
	Main Model	Control Group
1	Hardware/software failure (14.56%)	Energy consumption failure (15.07%)
2	Intentional attacks (13.87%)	Intentional attacks (13.79%)
3	Energy consumption failure (7.79%)	Hardware/software failure (13.35%)
4	Isolation failure (0)	Isolation failure (0)

## Data Availability

The data presented in this study are available on request from the corresponding author.
